# Field cricket genome reveals the footprint of recent, abrupt adaptation in the wild

**DOI:** 10.1002/evl3.148

**Published:** 2019-12-19

**Authors:** Sonia Pascoal, Judith E. Risse, Xiao Zhang, Mark Blaxter, Timothee Cezard, Richard J. Challis, Karim Gharbi, John Hunt, Sujai Kumar, Emma Langan, Xuan Liu, Jack G. Rayner, Michael G. Ritchie, Basten L. Snoek, Urmi Trivedi, Nathan W. Bailey

**Affiliations:** ^1^ Department of Zoology University of Cambridge Cambridge CB2 3EJ United Kingdom; ^2^ Division of Bioinformatics, Department of Plant Sciences Wageningen University & Research Wageningen 6708 PB The Netherlands; ^3^ Animal Ecology Netherlands Institute of Ecology Wageningen 6700 AB The Netherlands; ^4^ School of Biology University of St Andrews St Andrews Fife KY16 9TH United Kingdom; ^5^ Edinburgh Genomics University of Edinburgh Edinburgh EH9 3JT United Kingdom; ^6^ Institute of Evolutionary Biology University of Edinburgh Edinburgh EH9 3JT United Kingdom; ^7^ Earlham Institute Norwich Research Park Norwich NR4 7UZ United Kingdom; ^8^ School of Science and Health and the Hawkesbury Institute for the Environment Western Sydney University Penrith NSW 2751 Australia; ^9^ Centre for Ecology and Conservation University of Exeter Penryn TR10 9FE United Kingdom; ^10^ School of Environmental Sciences University of East Anglia Norwich NR4 7UZ United Kingdom; ^11^ Centre for Genomic Research University of Liverpool Liverpool L69 7ZB United Kingdom; ^12^ Theoretical Biology and Bioinformatics Utrecht University Utrecht 3584 CH The Netherlands; ^13^ Terrestrial Ecology Netherlands Institute of Ecology Wageningen 6700 AB The Netherlands

**Keywords:** Adaptation, feminization, genomics, rapid evolution, sexual signaling, trait loss

## Abstract

Evolutionary adaptation is generally thought to occur through incremental mutational steps, but large mutational leaps can occur during its early stages. These are challenging to study in nature due to the difficulty of observing new genetic variants as they arise and spread, but characterizing their genomic dynamics is important for understanding factors favoring rapid adaptation. Here, we report genomic consequences of recent, adaptive song loss in a Hawaiian population of field crickets (*Teleogryllus oceanicus*). A discrete genetic variant, *flatwing*, appeared and spread approximately 15 years ago. *Flatwing* erases sound‐producing veins on male wings. These silent flatwing males are protected from a lethal, eavesdropping parasitoid fly. We sequenced, assembled and annotated the cricket genome, produced a linkage map, and identified a *flatwing* quantitative trait locus covering a large region of the X chromosome. Gene expression profiling showed that *flatwing* is associated with extensive genome‐wide effects on embryonic gene expression. We found that flatwing male crickets express feminized chemical pheromones. This male feminizing effect, on a different sexual signaling modality, is genetically associated with the *flatwing* genotype. Our findings suggest that the early stages of evolutionary adaptation to extreme pressures can be accompanied by greater genomic and phenotypic disruption than previously appreciated, and highlight how abrupt adaptation might involve suites of traits that arise through pleiotropy or genomic hitchhiking.

**Impact Summary**

What are the genomic consequences of extremely rapid evolution in the wild? The adaptive evolutionary loss of male song in Hawaiian field crickets (*Teleogryllus oceanicus*) protects silent “flatwing” males from a lethal eavesdropping parasitoid fly, and invasion and spread of genetic variants causing silence was observed to occur over approximately 20 generations in a population on the island of Kauai and now appears to be fixed. To investigate the genomic and phenotypic consequences of this abrupt bout of adaptation, we first sequenced, assembled, and annotated the cricket genome – the first annotated reference genome for a field cricket. To provide a genomic resource for future work in crickets and allied taxa, we created a new, open‐access genome browser and database for crickets and katydids (http://www.chirpbase.org) and curated our data and scripts in it. Using RAD‐seq, we then constructed a high‐density linkage map for the species and found that the variant or variants causing flatwing are localized to a large region of the X chromosome, consistent with widespread genomic hitchiking. We performed gene expression analysis of embryonic crickets and found that flatwing is genetically associated with genome‐wide regulatory disruption during development. We quantified variation in another sexual signal, chemical pheromones, and discovered that flatwing is also strongly genetically associated with male pheromone feminization. Our findings illustrate how strong, widespread genetic and phenotypic effects can accompany the rapid emergence and spread of adaptive variants during the very earliest stages of rapid adaptation, and demonstrate how suites of traits that characterize alternative sexual polymorphisms might arise through pleiotropy or genomic hitchhiking following such genomic alteration.

Empirical studies have struggled to characterize genomic dynamics of the earliest stages of evolutionary adaptation in natural system, because it is difficult to detect new genetic variants at the moment they first appear and then spread in wild populations. However, understanding genomic causes and consequences of new adaptive mutations can help to identify and test factors that facilitate or inhibit rapid adaptation. For example, R. A. Fisher developed a “geometric” model that predicts adaptation should occur via mutations of small effect size, with impacts narrowly limited to a small number of phenotypic traits (Fisher 1930; Orr 2005; Bank et al. 2014). Later refinements to models of adaptation became more permissive of larger effect mutations, particularly during the earliest stages of adaptation under extreme selection (Kimura 1983; Orr 1998). However, questions remain about the extent to which novel adaptive variants of large effect are genetically associated with changes to other traits, altered gene expression, and potential loss of homeostasis, for example through pleiotropy or genomic hitchhiking (Nadeau et al. 2003). Here, we identified and characterized the genomic signature of very recent sexual signal loss in Hawaiian field crickets, *Teleogryllus oceanicus*, and tested the associated genetic consequences of this rapid adaptation for a different sexual signal, chemical pheromones.

Male crickets sing to attract and court females and to fight with rivals, but approximately 16 years ago, silent *T. oceanicus* males were detected in populations on the Hawaiian archipelago (Zuk et al. 2006, 2018) (Fig. 1A). They spread rapidly. First observed in 2003 in a population on Kauai, where they were previously not observed, silent male crickets rapidly spread in fewer than 20 generations (with three to four generations per year) to near‐fixation under selection imposed by a lethal parasitoid fly, *Ormia ochracea* (Fig. 1B) (Zuk et al. 2006). Female flies acoustically locate male crickets by eavesdropping on their songs, but silent flatwing males have feminized wings lacking typical male structures used to produce sound and are thus protected (Fig. 1C). The genetic mutation(s) underlying the flatwing phenotype have been shown previously using standard genetic crosses to follow discrete segregation patterns. Sex determination is XX/XO (female/male), and flatwing's sex‐linked, male limited expression indicates it is a variant, or cluster of closely linked variants, that segregate in the manner of a single‐locus located on the X chromosome (Tinghitella 2008; Pascoal et al. 2014). The morph has been observed emerging in parasitized populations on other Hawaiian islands, and in at least one case appears to be caused by distinct genetic mechanisms (Pascoal et al. 2014; Zuk et al. 2018). The genetic loss of male song in the Kauai population is a canonical example of rapid evolution in the wild (Dugatkin 2008), and all males in this population now appear to be flatwing (Rayner et al. 2019a). Nevertheless, the continued existence of the population indicates that silent males still find mates and must compensate for their inability to sing. The selective environment promoting the rapid spread of flatwing crickets is understood, but the genomic causes and consequences of this rapid evolutionary event remain open questions. Flatwing males have distinctly feminized wings and cannot produce sexual signals critical for reproductive fitness: how did such a spectacularly disruptive phenotypic change invade the genome of crickets so quickly?

**Figure 1:**
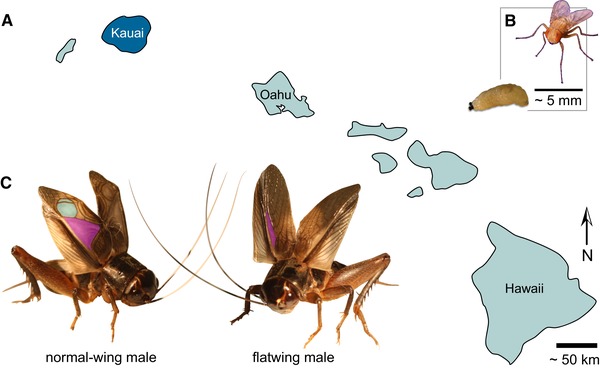
Evolutionary loss of song in Hawaiian crickets. (A) The field cricket *T. oceanicus* is thought to have migrated to the Hawaiian archipelago from other islands in Oceania, and is attacked by the fatal, acoustically‐orienting parasitoid fly *Ormia ochracea* on Kauai, Oahu, and Hawaii. We studied crickets from a population in Kauai, highlighted in dark blue, where parasitoid infestation rates have historically been highest. (B) Adult female parasitoid fly and mature fly larva. Gravid female flies locate hosts by eavesdropping on singing male crickets, then they eject larvae that burrow into the host and consume its viscera before emerging to pupate. Infestation is fatal, and the flies exert significant natural selection against male song. (C) Normal‐wing males (left) of this field cricket species produce advertisement, courtship, and aggressive songs by elevating and rubbing together forewings that bear specialized sound‐producing venation. A toothed file on the right wing engages with a thickened ridge of tissue on the opposite, causing resonators to vibrate and produce sound. Two principal resonators are highlighted on this male's right forewing: the harp in purple and the mirror in turquoise. Flatwing males (right) have wings that are feminized and lack, or have severely reduced, resonators. They still make wing motions characteristic of singing despite the structural inability to produce sound (Schneider et al. 2018), but their silence protects them from the fly (Zuk et al. 2006). Currently, 100% of males from the population studied on Kauai exhibit flatwing morphology. (Photo credits: N.W. Bailey)

## Materials and Methods

### CRICKET REARING AND MAINTENANCE

Laboratory stocks of *T. oceanicus* were originally derived from the population in which the flatwing phenotype was first observed on Kauai (Zuk et al. 2006), and a population near Daintree, Australia (Pascoal et al. 2016b), which contains no flatwing crickets. Stocks were maintained in 16‐L plastic containers containing cardboard egg cartons for shelter. All crickets were reared in a single, temperature‐controlled chamber at 25°C, on a 12:12 light:dark cycle. They were maintained regularly and fed *ad libitum* with Excel Junior and Dwarf rabbit pellets (Burgess) and provided water in a moist cotton pad that also served as ovipositor substrate.

### GENOME SEQUENCING

Three Illumina sequencing libraries (paired‐end TruSeq libraries with insert sizes of 180, 300, and 600 bp) were prepared at Edinburgh Genomics. Genomic DNA (gDNA) was extracted from the head capsule and muscle tissue of a single *T. oceanicus* female sourced from the Kauai stock population using a DNeasy Blood & Tissue kit (Qiagen). DNA was quality‐checked using Nanodrop and Qubit. We supplemented reads from the above libraries with additional sequences from two TruSeq Nano Pippin selected libraries with insert sizes of 350 bp and 550 bp, one 8 kb Nextera gel‐plus mate‐pair library, and 1 PacBio library. For these four libraries, gDNA from a separate, single female cricket from the same laboratory population was extracted using a high molecular weight Genera Puregene Cell Kit (Qiagen). The TruSeq libraries were sequenced on five lanes of an Illumina HiSeq 2000 v3 to yield 100 bp paired‐end reads. NanoPippin libraries and the Nextera mate‐pair library were sequenced on 2 Illumina HiSeq 2500 lanes to yield 250 bp paired‐end reads. The PacBio library was constructed by purifying the extraction with 1x AMPure beads (Agencourt). DNA quality was checked using Nanodrop and Qubit. Average DNA size and degradation was assessed using a high sensitivity genomic kit on a fragment analyzer. Size‐selected and non‐size‐selected libraries were made by shearing gDNA using g‐TUBEs (Covaris). Size selection was performed using the BluePippin DNA Size Selection System with 0.75% cassettes and cutoffs between 7 and 20 kb. Preparation of both libraries then proceeded using the same protocol. We treated DNA for 15 minutes at 37°C with Exonuclease V11. DNA ends were repaired by incubating for 20 minutes at 37°C with Pacific Biosciences damage repair mix. Samples were then incubated with end repair mix for 5 minutes at 25°C followed by washing with 0.5x AMPure and 70% ethanol. DNA adapters were ligated overnight at 25°C. Incubation at 65°C for 10 minutes was used to terminate ligation reactions, and then samples were treated with exonuclease for 1 hour at 37°C. We purified the SMRTbell library using 0.5x AMPure beads and checked quality and quantity using Qubit. Average fragment size was quantified using a fragment analyzer. For sequencing, primers were annealed to the SMRTbell library at values determined using PacBio's Binding Calculator. A complex was formed using DNA polymerase (P6/C4 chemistry), bound to MagBeads, and then used to set up 43 SMRT cells for sequencing to achieve 10X coverage. Sequencing was performed using 240‐minute movie times.

### GENOME ASSEMBLY

Raw reads from all Illumina libraries were trimmed using cutadapt version 1.8.3 (Martin 2011) to remove adapters, primers and poor quality bases, and then error‐corrected using BLESS version 1p02 (Heo et al. 2014). PacBio reads <1000 bp were discarded. The original fragment length of the 350 bp library was shorter than the sequenced paired read length of 500 bp, so reads from this library were merged using Vsearch version 1.10.1 (Rognes et al. 2016). Platanus version 1.2.4 (Kajitani et al. 2014) was used to assemble error‐corrected reads from all Illumina libraries except the mate‐pair library; reads from the latter were added at the scaffolding stage. Next, we selected the contigs >1000 bp and combined these with the PacBio data to generate a hybrid assembly using PBJelly version 15.2.20 (English et al. 2012). Pilon version 2.1 (Walker et al. 2014) was used to improve local base accuracy, and BUSCO version 2.1 (Simao et al. 2015) was used to assess genome quality through gene completeness.

### REPEAT ANNOTATION

We used *de novo* and homology‐based approaches to identify repetitive regions. We first built a *de novo* repeat library using RepeatModeler version 1.0.10 (Tarailo‐Graovac and Chen 2009), with dependencies RECON version 1.08 and RepeatScout version 1.0.5 (Price et al. 2005). To scan and classify interspersed repeats and low complexity DNA sequences at the DNA level, we searched the cricket genome sequence against the Dfam consensus database (20170127) (Hubley et al. 2016), RepBase (20170127) (Bao et al. 2015), and the *de novo* repeat library using RMBlast version 2.6.0+ (Boratyn et al. 2012) and RepeatMasker version 4.0.7 (Smit et al. 2013–2015). Protein‐level repeats were identified by searching against the TE Protein Database using RepeatProteinMask version 4.0.7 (Smit et al. 2013–2015). Unclassified repetitive elements were further classified by TEclass version 2.1.3 (Abrusan et al. 2009), a programme using a support vector machine learning algorithm. Tandem repeats were also identified in the cricket genome using Tandem Repeat Finder version 4.09 (Benson 1999).

### GENE PREDICTION

Before running gene prediction pipelines, repetitive regions identified above were masked using an in‐house Perl script. We built a pipeline including *ab initio*, homology and transcriptome‐based methods to predict protein‐coding genes in the cricket genome (Fig. S1). For *ab initio* prediction, SNAP 2013‐11‐29 (Korf 2004), Glimmer‐HMM version 3.0.4 (Majoros et al. 2004), GENEID version 1.3 (Blanco et al. 2007), and BRAKER version 2.0.4 (Hoff et al. 2016) were used to generate preliminary gene sets from the repeat‐masked genome. Specifically, reads obtained from the *T. oceanicus* transcriptome were aligned against the repeat masked genome with TopHat2 version 2.0.10 (Kim et al. 2013). SAMTOOLS version 0.1.19 (Li et al. 2009) was used to sort and index the resulting Binary Alignment Map (BAM) format file. This BAM file was processed in BRAKER version 2.0.4 (Hoff et al. 2016), which used transcriptome data to train GENEMARK‐ET version 4.33 (Lomsadze et al. 2014), generate initial gene structures, and then subsequently train AUGUSTUS version 3.2.2 (Stanke et al. 2008) and finally integrate RNA‐seq information into final gene predictions. For other *ab initio* gene prediction programmes, gene sets from *Locusta migratoria* (Wang et al. 2014), *Acyrthosipon pisum* (International Aphid Genomics Consortium 2010), and *Drosophila melanogaster* (Gramates et al. 2017) were used for model training. For homology‐based prediction, we aligned protein sequences of five insect species (*L. migratoria*, Wang et al. 2014; *Drosophila melanogaster and Anoplophora glabripennis*, McKenna et al. 2016; *Nilaparvata lugens*, Xue et al. 2014; and *Cimex lectularius*, Benoit et al. 2016) to the repeat‐masked cricket genome using TBLASTN version 2.2.26 (*E* < 10^−5^). The boundaries of potential genes were further identified using BLAST2GENE version 17 (Suyama et al. 2004). We then ran GENEWISE2 2‐4‐1 (Birney et al. 2004) to obtain accurate spliced alignments and generate a final, homology‐based gene set. For prediction based on transcriptome data, a *de novo T. oceanicus* transcriptome assembly generated for a separate study (Rayner et al. 2019b) using Trinity version 2.2.0 (Grabherr et al. 2011) was filtered based on gene expression level, and then passed to Program to Assemble Spliced Alignments (PASA version 2.2.0) (Xu et al. 2006). PASA performed transcript alignments to the cricket genome, generated a new transcript assembly, and predicted gene structures. All *ab initio*, homology, and transcriptome‐based gene sets were then combined into a weighted consensus set using EVidenceModeler (EVM r2012‐06‐25) (Haas et al. 2008). We removed genes likely to be spurious, those with low EVM support, partial genes with coding lengths shorter than 150 bp, and genes only supported by a minority (≤2) of *ab initio* methods (Yang et al. 2017). PASA was used to further update the filtered consensus gene set to produce a finalized official gene set. The completeness of this final gene set was assessed by both BUSCO version 2.1 (using the arthropoda dataset) (Simao et al. 2015) and transcriptome data.

### FUNCTIONAL ASSIGNMENT

Putative gene functions were assigned using InterPro (InterProScan 5) (Finn et al. 2017), SwissProt (February 2018) (Bairoch and Apweiler 2000), TrEMBL (February 2018) (Bairoch and Apweiler 2000), and RefSeq nonredundant (NR) protein (106,376,657 sequences) and Kyoto Encyclopedia of Genes and Genomes (KEGG) gene (family_eukaryotes) databases. Briefly, we first obtained protein sequences from the final gene set using EVM r2012‐06‐25 (Haas et al. 2008). Functional annotation and gene ontology (GO) terms were assigned to genes based on protein sequence using InterProScan 5 (Jones et al. 2014). These proteins were also blasted against SwissProt, TrEMBL and NR databases (BLASTP, *E* < 10^−5^), and assigned their best hits as functional annotations. GO terms were assigned using GO annotations downloaded (March 26, 2018) from the GO Consortium (Adams et al. 2000; The Gene Ontology Consortium 2017). BLAST2GO (unix_4_1_x54) (Gotz et al. 2008) was implemented to further assign unassigned genes using NCBI databases, and KEGG Orthology (KO) terms were assigned using BlastKOALA version 2.1 (Kanehisa et al. 2016b).

### GENOME ANCHORING

ALLMAPS version 0.7.7 (Tang et al. 2015) was used to detect chimeric scaffolds, anchor the cricket genome to the linkage map (see below), and construct pseudo‐molecules (reconstructed portions of chromosomal sequence). We first built a consensus genetic map based on male and female genetic distances obtained from linkage maps, in which equal weighting was applied for both sexes. Then, scaffolds for which more than four markers mapped to multiple linkage groups were designated as chimeric scaffolds and split. After this correction was applied, scaffolds anchored to the linkage maps were oriented and ordered based on the consensus genetic map. We used a custom Perl script to order unanchored scaffolds according to their length, and liftOver (March 2018) (Kent et al. 2002) to convert genome coordinates based on anchoring results.

### ChirpBase—A GENOME BROWSER AND DATABASE

We created ChirpBase, an open‐access community genomics resource for singing insects such as field crickets and katydids. It can be accessed at http://www.chirpbase.org where users may view and download genomic data and scripts presented in this study in addition to uploading data. An index page links to an Ensembl page, where assembly statistics can be visualized using a Challis plot or compared in tabular format. A plot illustrating codon usage is presented, and BUSCO scores can be visualized. Additional linking pages include a basic local alignment search tool (BLAST) page and a download page for accessing raw data. We used the GenomeHubs framework (Challis et al. 2017) to set up ChirpBase. The database is hosted using a Linux container (LXC) on a remote computer, linked to a cluster via an intermediate import computer. A MySQL docker container was started in the LXC, where a database *ini* file resided to guide additions to the database. An Ensembl‐easy mirror Docker container was run to import the database into the MySQL container, uploading data designated in the *ini* file from the LXC to the database. The MySQL container links to an Ensembl EasyMirror container, BLAST container, and a download container.

### LINKAGE AND QUANTITATIVE TRAIT LOCUS MAPPING CROSSES

We constructed a linkage map for *T. oceanicus* crosses designed to maximize recombination on the X chromosome by retaining only families where *flatwing*‐carrying and *normal‐wing*‐carrying X chromosomes were present together in dams, as the X is only diploid in females (Fig. S2), combined with restriction‐site associated DNA sequencing (RAD‐seq) to identify markers. *Flatwing* segregates on the X chromosome (Tinghitella 2008; Pascoal et al. 2014), so mapping was performed with F_3_ offspring to increase recombination on the X. We set up two parental mapping families by crossing a flatwing sire from the Kauai stock line with a virgin dam from the Daintree, Australia stock line. Daintree females were used to maximize our opportunity to genetically map segregating variation in other phenotypes. Female F_1_ offspring from parental crosses were heterozygous for *flatwing*, enabling recombination on the X. Full‐sib matings were then performed with F_1_ males, all of which were normal‐wing. The resulting F_2_ female offspring were a mix of homozygous *normal‐wing* genotypes on the X, or heterozygous with respect to wing morph. Recombination between *flatwing* and *normal‐wing* genotypes was similarly possible in the heterozygous F_2_ females, but their phenotype is not externally detectable. We then mated F_2_ females with full‐sib *flatwing* males from the same generation. Screening male morph types in the resulting F_3_ offspring enabled us to identify F_2_ crosses involving heterozygous females, as all male offspring of homozygous *normal‐wing* females expressed normal‐wing morphology. The crossing procedure resulted in 10 F_3_ mapping families from the original two parental families, from which a total of 192 females, 113 normal‐wing males, and 86 flatwing males were used for RAD‐seq analysis (below).

### MARKER IDENTIFICATION USING RAD‐SEQ

RAD‐seq was used to identify single nucleotide polymorphisms (SNPs) in F_3_ offspring (*n* = 391), P_0_ dams and sires (*n* = 4), and the F_2_ sires and dams (*n* = 19) that were used to produce mapping individuals in the F_3_ generation. For each individual, gDNA extraction and quality control was performed as described above prior to library preparation. gDNA was digested using SbfI (New England BioLabs). We barcoded individuals by ligating P1 adapters (8 nM), then sheared and size selected 300–700 bp fragments. After ligating P2 adapters to sheared ends, parents were sequenced to an average coverage of 120× and offspring to 30× on an Illumina HiSeq 2000.

### LINKAGE MAP CONSTRUCTION

Reads from all paired end RAD libraries were demultiplexed by sample using process_radtags from Stacks version 1.46 (Catchen et al. 2013), mapped against the reference genome assembly using BWA‐MEM version 0.7.15‐r1140 (Li and Durbin 2009) and duplicates marked using PicardTools MarkDuplicates version 2.9.2 (http://broadinstitute.github.io/picard). Variants were called using samtools mpileup (version 1.3, parameters ‐d 2000 ‐t DP,DPR,DV,DP4,SP ‐Aef ‐gu) and bcftools call (version 1.3, parameters ‐vmO z ‐f GQ). The resulting variants were filtered using vcfutils.pl (included with bcftools) with minimum quality 50 and a minimum read depth of 150 (‐Q 50 ‐d 150) to only retain high‐quality variants. The vcf format was converted to the required Lep‐MAP2 input format using a custom script of the RADmapper pipeline (RAD_vcf_to_lepmap_with_sexmarker_conversion.py, https://github.com/EdinburghGenomics/RADmapper). During this conversion samples that did not fit initial relatedness expectations (*n* = 8, using vcftools relatedness2 and visual inspection of a heatmap) and samples from family I (which lacked a genotyped father, *n* = 59) and P0 parents (*n* = 4) were excluded from linkage map creation. Putative X‐linked markers (male_het ≤ 1, female_het > 20, het_sire ≤ 1) were converted to biallelic markers in the relevant male offspring and sires using a dummy allele (Table S1). The linkage map was then created using the following steps and parameters in Lep‐MAP2 version 0.2 (Rastas et al. 2015) (Filtering: dataTolerance 0.05 keepAlleles = 1; SeparateChromosomes: losLimit = 10 sizeLimit = 10 informativeMask = 3; JoinSingles: lodLimit = 5; OrderMarkers: filterWindow = 10 polishWindow = 100; OrderMarkers evaluateOrder: filterWindow = 10 polishWindow = 100). The resulting linkage map files were merged with the marker and sample information using a custom script from the RADmapper pipeline (LG_to_marker.py).

### QUANTITATIVE TRAIT LOCUS MAPPING

To identify the flatwing locus on the putative X chromosome (LG1), we performed analysis of variance (ANOVA) for each marker using the lm package in R (version 3.1) and 178 male samples (105 normal‐wing + 73 flatwing; as above excluding all grandparental, parental and female samples together with samples that clustered with the wrong family, had insufficient coverage to calculate relatedness or did not have cuticular hydrocarbon [CHC] data, see below). Individual *P*‐values were corrected to account for multiple testing using Bonferroni correction and markers supported by a log‐of‐odds (LOD)10 cutoff were plotted. Quantitative trait locus (QTL) for all 26 CHC peaks as well as the principle components from the CHC analysis were mapped to the linkage groups using mixed linear models in ASReml version 4. Mapping used a GWAS‐type approach, taking into account genetic relatedness between individuals (Calus 2010). The markers mapped to the autosomal linkage groups 2–19 were filtered to contain only biallelic SNP markers with a MAF ≤ 0.01 and <5% missing samples per marker. Only male samples were selected (the same *n* = 178 as for mapping *flatwing* above), as our aim was to map male CHCs (not sex‐related associations) on the putative X (LG1) and autosomes using principle components from the CHC analysis as well as individual compounds as traits. The remaining 21,047 markers were used to calculate pairwise genetic relatedness with the first normalization (VanRaden 2008). The resulting inverse relatedness matrix was used as random effect in a model: CHC trait ∼ mu marker r! Giv(animal). *P*‐values for all markers were extracted from the results and corrected for multiple testing using Bonferroni correction. The same model was used to assess LG1 separately with the same set of samples, for which 6537 markers were used after filtering.

### PURE‐BREEDING LINES AND EMBRYO SAMPLING FOR RNA‐SEQ

Kauai lines homozygous for the *flatwing* and *normal‐wing* genotypes were used for examining differential gene expression. Their establishment has been described previously (Pascoal et al. 2016a). Briefly, one generation of crosses was performed, starting with the laboratory population derived from Kauai and crossing males of either wing phenotype to virgin females of unknown genotype. Because the phenotypic effects of *flatwing* are sex‐limited, family‐level screening of the resulting male offspring was performed to select homozygous *flatwing* and homozygous *normal‐wing* lines, resulting in a final selection of three pure‐breeding lines for each morph genotype. Developing embryos were sampled from eggs laid by females from each line. Females were maintained in laboratory culture as above, and their oviposition substrates were monitored. Eggs were removed from the substrate and immediately preserved in 500 µL of RNAlater (Qiagen) at the stage when eye pigmentation first develops, ca. two weeks after laying. This time point corresponds approximately to embryonic stage 13–14 in the related grylline species *Gryllus bimaculatus* (Donoughe and Extavour 2016). After removing the outer egg chorion, the thoracic segment of each nymph was microdissected. Nymphs cannot be sexed based on external morphology until a later stage of juvenile development, and as chromosomal sex determination is XX/XO, screening for sex‐specific markers is not possible. To minimize potential variation in sex ratio of samples between lines, and ensure a sufficient volume of tissue to extract RNA, thoracic tissue from *n* = 8 nymphs was pooled for each replicate, and six biological replicates were produced for each morph type (two per line).

### RNA‐SEQ AND GENE EXPRESSION PROFILING

Total RNA was extracted using the TRIzol plus RNA purification kit (Life Technologies) and DNAse treated using PureLink (Invitrogen). RNA was quantified and quality checked using Qubit assessment (Invitrogen) and Bioanalyser RNA Nano Chips (Agilent), respectively. To isolate mRNA we depleted samples with RiboZero. After verifying depletion, cDNA libraries were constructed using the ScriptSeq protocol (Epicentre) with AMPure XP beads for purification. Following barcoding and multiplexing, final quality was checked and qPCR performed using Illumina's Library Quantification Kit (Kapa). Sequencing was performed on an Illumina HiSeq 2000 version 3, with 1% PhiX DNA spike‐in controls to produce 100 base paired‐end reads. CASAVA version 1.8.2 was used to demultiplex reads and produce raw fastq files, which were then processed with Cutadapt version 1.2.1 (Martin 2011) and Sickle version 1.200 (Joshi and Fass 2011) to remove adaptor sequences and trim low‐quality bases. Further quality assessment was performed in FastQC. Expression analysis of RNA‐seq data was performed broadly following the protocol published by Pertea et al. (2016). Reads were aligned to the genome using HISAT2 version 2.1.0 with strand‐specific settings, and transcripts compiled for each sample in StringTie version 1.3.4, using the gene annotation file as a reference, which were then merged across all samples to produce a single annotated reference transcriptome. Sample transcript abundances were estimated with the parameter ‐e specified to restrict abundance estimation to annotated transcripts. Differential expression analysis was performed at the gene level following normalization of counts by trimmed mean of *M*‐values, using a generalized linear model with negative binomial distribution and a single predictor variable of “morph” in the edgeR version 3.20.9 package (Robinson et al. 2010) in R version 3.4.1. Only genes with an expression level greater than 1 count per million in at least three samples were included in the analysis. Significance‐testing was performed using likelihood ratio tests, and genes were considered significantly differentially expressed (DE) between morph genotypes if false‐discovery rate (FDR)‐adjusted *P*‐values were below a threshold of 0.05.

### SCREENING FOR TOP CANDIDATE GENES ASSOCIATED WITH FLATWING

We adjusted *P*‐values for significant marker associations in the flatwing QTL mapping procedure using Bonferroni correction with a cut‐off of *P* < 0.001. Three sources of information were used to comprehensively and robustly detect a set of top candidate genes associated with the flatwing phenotype. We detected genes (i.e. any overlapping portion of a predicted gene sequence) located in 1 kb flanking regions of all significant QTL markers after FDR correction as above, and defined these as QTL‐associated candidates. We then subsetted these genes to retain only those located in the 1 kb flanking regions of the most significant (top 1%) of all QTL markers, and defined these as Top 1%‐associated candidates. We also obtained the flatwing‐associated sequences from a previously published bulk segregant analysis (BSA) of Kauai flatwings (Pascoal et al. 2014), and defined the BSA reference sequences containing flatwing‐associated SNPs as flatwing‐associated BSA sequences. We mapped these BSA sequences to the *T. oceanicus* reference genome using BWA‐MEM with default parameters (Li and Durbin 2009). Coordinates of mapped sequences were extracted from the resulting BAM files using SAMTOOLS (Li et al. 2009) and custom Perl scripts, and we only retained those sequences that were anchored to LG1. Genes within 1 kb of these retained sequences were defined as BSA‐associated candidates. Finally, we extracted DE genes from the embryonic thoracic transcriptome analysis above, and defined these as DEG‐associated candidates. To ensure a reliable final candidate gene set for flatwing, we only retained genes supported by at least two of these four gene sets. We used KEGG pathway mapping (color pathway) to reconstruct pathways and obtain reference pathway IDs (Kanehisa et al. 2016a). To characterize significantly enriched GO terms and KEGG pathways in DEGs, we implemented the hypergeometric test in enrichment analyses. *P* values for each GO and KEGG map term were calculated and FDR‐adjusted in R.

### CHC EXTRACTION AND GAS CHROMATOGRAPHY–MASS SPECTROMETRY

We extracted CHCs from 394 individuals from the F_3_ mapping generation prior to extracting gDNA for RAD‐seq. Extraction and analysis of CHCs followed previous methodology (Pascoal et al. 2016b), which is briefly described here. Subjects were flash‐frozen for several minutes at −20°C and then thawed. They were individually placed into 4 mL borosilicate glass vials (QMX Laboratories) and immersed for 5 minutes in 4 mL of HPLC‐grade hexane (Fisher Scientific), then removed from the vials and stored for later processing. We evaporated a 100 µL aliquot of each sample overnight in a 300 µL autosampler vial (Fisher Scientific). CHC extracts were transported to the University of Exeter for gas chromatography mass spectrometry (GC/MS) using an Agilent 7890 GC linked to an Agilent 5975B MS. Extracts were reconstituted in 100 µL of hexane with a 10 ppm pentadecane internal standard, and 2 µL of this was injected into the GC/MS using a CTC PAL autosampler at 5°C. The carrier gas was helium and we used DB‐WAX columns with a 30 m × 0.25 mm internal diameter and 0.25 µm film. Injection was performed in split‐less mode. The column profile was optimized for separation of the CHC extract (Pascoal et al. 2016b) to start at 50°C for 1 minute, followed by a temperature ramp of 20°C per minute until finally holding at 250°C for a total run time of 90 minutes. The inlet temperature was 250°C and the MS transfer line was 230°C. We recorded electron‐impact mass spectra using a 70 eV ionization voltage at 230°C, and a C_7_‐C_40_ alkane standard was run as a standard to enable the later calculation of peak retention indices.

### QUANTIFICATION AND ANALYSIS OF CHC PROFILES

For each individual, we used MSD CHEMSTATION software (version E.02.00.493) to integrate the area under each of 26 CHC peaks (Table S2) following (Pascoal et al. 2016b). Peak abundances were standardized using the internal pentadecane standard and log_10_ transformed prior to analysis. After accounting for samples that failed during extraction or during the GC run (*n* = 10), labeling error (*n* = 1), and one normal‐wing male CHC profile that was identified as an outlier and removed during analysis (Fig. S3), we analyzed a total of *n* = 86 flatwing males, *n* = 112 normal‐wing males, and *n* = 185 females of unknown genotype. To test whether CHC profiles differed between males of either wing morph, we first performed dimension reduction using principal components analysis (PCA) on male data only. JMP Trial version 14.1.0 (SAS Institute Inc.) was used to draw a 3D scatterplot of the first three PCs. To assess statistical significance, we performed a multivariate analysis of variance using all principal components (PC) with eigenvalue > 1.00 (*n* = 6). This indicated a highly significant difference among male morphs that formed the basis of QTL mapping described above. To visualize the difference between flatwing and normal‐wing male CHC profiles with respect to female CHC profiles, we performed a discriminant function analysis (DFA) for all samples and all 26 peaks. DFA highlights the maximal difference between predefined groups, with maximum group differences indicated by the first DF axis. Statistical analyses of CHC data were done in SPSS (version 23).

## Results and Discussion

### SEQUENCING THE CRICKET GENOME AND MAPPING FLATWING

**Figure 2:**
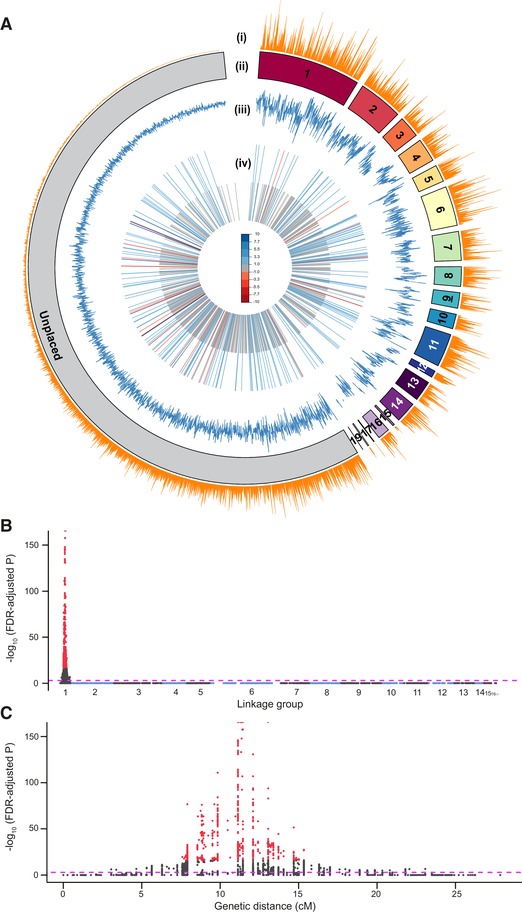
*Teleogryllus oceanicus* genome and regions associated with the flatwing phenotype. (A) Circos plot providing an overview of the genome. Linkage groups (LGs) upon which genome scaffolds were anchored are shown in different colors, with unplaced scaffolds in gray. LG1 was identified as the X chromosome based on heterozygosity and coverage filters (see main text). Tracks: (i) gene density, (ii) linkage group pseudomolecules, (iii) transposable element density, (iv) genes differentially expressed (DE) in the thoracic tissues of embryos homozygous for *flatwing* versus *normal‐wing* genotypes. Longer bars are DE genes for which log_2_ fold‐change >1 between genotypes, and short gray bars are all other DE genes. Colors indicate the magnitude of upregulation (red) versus downregulation (blue) in *flatwing* compared to *normal‐wing* embryos. (B) Genome‐wide Manhattan plot of the flatwing QTL. Alternating shades of gray and blue indicate different LGs. The horizontal dashed line indicates an FDR‐corrected significance threshold of (*P* < 0.001), and the top 1% most significant QTL markers are plotted in red. (C) Enlarged plot for LG1 (X chromosome) showing the flatwing‐associated peak.

We studied the genomic signature of song loss in the Kauai population where flatwing crickets were first discovered, and in which rapid spread has been most thoroughly documented (Zuk et al. 2006). Using females from laboratory stock, we sequenced the *T. oceanicus* genome and generated an assembly of 2.045 Gb consistent with flow cytometry size estimates (Pascoal et al. 2014), with a scaffold N50 of 62.6 kb (Table S3). We established an F_3_ mapping population using crosses designed to maximize recombination on the X chromosome (Fig. S2). Mapping offspring and parents were sequenced using RADseq, and a map was assembled containing 19 linkage groups. We identified linkage group 1 (LG1) as the X chromosome by applying coverage and heterozygosity filters and dummy coding putative X‐markers prior to constructing the map. LG1 was the largest linkage group, with a female recombination length of 379 cM and a male length of 195 cM (Fig. S4). After resolving chimeric scaffolds (Table S4), 35.6% of the genome was anchored to a linkage map using a LOD5 cutoff (Fig. 2A and Table S5). *Teleogryllus oceanicus* has a haploid chromosome number of (13 + X), so the additional five linkage groups likely correspond to unjoined chromosomal segments.

We performed gene prediction and annotation using custom pipelines incorporating *ab initio*, homology, and transcriptome‐based approaches (Fig. S1). Evidence from different gene prediction and annotation methods was weighted and filtered to predict a final, conservative set of 19,157 genes, 75% of which had functional annotation (Table S6 and Fig. S5). Gene density was assessed (Fig. 2A, track i), and we tested whether the putative X linkage group showed a different distribution of repeat content relative to the other linkage groups, across eight common categories of repeats. It did not (Fig. 2A, track iii; Table S7; Fig. S6). *Teleogryllus oceanicus* gene features were compared to 10 other insect species (Table S8), and we contrasted transposable element classifications with three other recently published insect genomes (Table S9). The *T. oceanicus* genome and metadata associated with it are curated in ChirpBase (http://www.chirpbase.org), a GenomeHubs Ensembl genome browser (Challis et al. 2017) that we created as an openly available, community‐based genomics resource for researchers working on singing insects.

*Flatwing* was definitively mapped to the putative X chromosome using markers supported by a LOD10 cutoff and a mixed model ANOVA‐based approach designed to control for uneven genomic relatedness caused by family structure in the mapping crosses (Fig. 2B; no other linkage group had markers showing associations exceeding the genome‐wide significance threshold of *P* < 0.001). To cope with the particularly high marker association on the putative X chromosome caused by the discrete mode of inheritance of *flatwing* and the different effective population size of the X compared to autosomes, we identified the QTL using only the top 1% of markers after FDR correction, yielding a prominent peak occupying approximately one‐third of the X chromosome (Fig. 2C).

### REGULATORY CONSEQUENCES ASSOCIATED WITH *FLATWING*

Flatwing morphology is observable in males during mid‐ to late‐instar stages of juvenile development, so we examined early embryonic gene expression differences associated with *flatwing*. Females carrying the genotype cannot be visually distinguished and embryos cannot be readily sexed, so we used replicate laboratory lines homozygous for *flatwing* or *normal‐wing* genotypes to detect widespread differential gene expression in the developing thoraces of embryonic crickets. We found 830 genes DE, 204 of which had a log_2_ fold‐change >1, and a predominant pattern of downregulation in *flatwing* crickets (Fig. 2A, track iv; Table S10; Fig. S7). DE genes associated with *flatwing* were widely distributed across linkage groups and unmapped scaffolds (Fig. 2A, track iv).

These physically dispersed expression effects are consistent with a scenario in which *flatwing* acts as a master regulatory switch during early development, with a broad cascade of downstream effects. Pathways reconstructed using differential expression data are consistent with a master regulatory switch. For example, adherens junction activity was enriched, which affects epithelial patterning during early development (Tables S11 and S12). Using a stringent and redundant approach combining information from gene sets identified in the QTL study, RNA‐seq experiment and a previously published bulked segregant analysis (Pascoal et al. 2014), we identified 51 annotated protein‐coding genes located within LG1 as top flatwing‐associated candidates (Table S13). GO enrichment analysis indicated that *positive regulation of developmental process* was overrepresented in this candidate gene set, with three genes in particular (*NBL1, GOGA4, UNC89*) known to play a fundamental role in the regulation of cell differentiation (Table S14). However, it is plausible that loci hitchhiking with the causal genetic variant(s) underlying the flatwing phenotype also have regulatory effects. Such joint effects could compound gene regulatory consequences of novel adaptive variants.

### CANDIDATE GENE DISCOVERY

In most pterygote insects, wings are derived from imaginal discs formed during development by the invagination of embryonic ectoderm (Snodgrass 1993). Previous work mainly in *Drosophila melanogaster* has established that the developmental elaboration of wing venation patterns requires the involvement of numerous transcription factors and complex coordination across numerous signaling pathways (De Celis 2003). Here, we found that seven of 51 flatwing associated candidate genes have reported involvement in wing development in *D. melanogaster*. For example, *Collier* encodes a transcription factor required for wing disc patterning (Vervoort et al. 1999), and *Myoglianin* expression is required for normal wing disc development (Hevia and de Celis 2013). *ROR1* encodes a transmembrane tyrosine‐protein kinase receptor involved in phosphorylating MAP kinases (Bicocca et al. 2012), and reduction of MAPK activity through *ROR1* silencing can lead to a loss of wing venation phenotype (De Celis 2003). The protein krasavietz is encoded by *PKRA*, and establishes planar cell polarity in the wing (Carvajal‐Gonzalez et al. 2016), disruption of which can lead to wing distortion (Adler 2012). Knockouts and mutants in *Pelle*, *Gcn5*, and *Plexin‐A4* show wing shape and venation alterations with features similar to flatwing (Carre et al. 2005; Wu et al. 2015; Okada et al. 2016).

### GENETICALLY ASSOCIATED FEMINISATION OF MALE PHEROMONES

**Figure 3:**
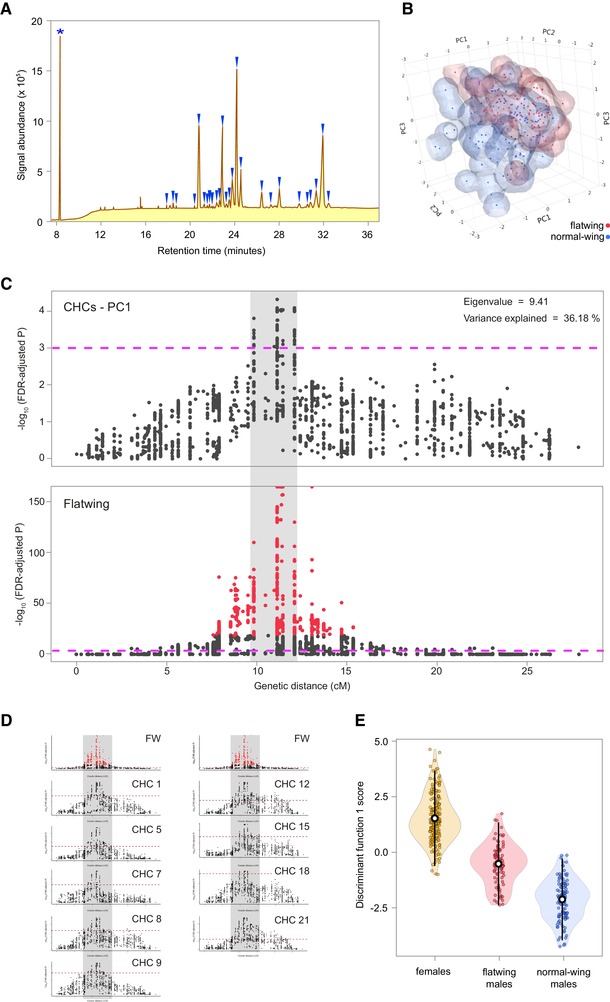
Genetic colocalization of the flatwing phenotype and male chemical pheromone feminization. (A) Diagram of a *T. oceanicus* cuticular hydrocarbon (CHC) chromatogram, with the 26 measured peaks indicated by blue wedges. The asterisk indicates the internal standard (pentadecane). (B) Space‐filling scatterplot of the first three principal components describing male CHC profiles, illustrating differences between flatwing and normal‐wing males (variance explained for PC1: 35.18%, PC2: 10.14%, PC3: 9.58%). (C) Comparison of QTL on the putative X chromosome for CHCs (top; first principal component mapped) and flatwing (bottom, same as Fig. 2C). Gray shading indicates the extent (in cM) of the CHC peak, showing overlap with the flatwing QTL. Dashed lines indicate FDR‐corrected significance of *P* < 0.001, and red line points the top 1% significant flatwing QTL markers. Note the different *y*‐axis scales. (D) Univariate analyses revealed nine individual CHC components, which also co‐localized with flatwing. The original flatwing QTL is plotted at the top of each column. Gray shading spans the genetic region of co‐localization. Numbers refer to compounds indicated in *A*, and dashed lines indicate an FDR‐corrected significance threshold of *P* < 0.001. (E) Discriminant function scores describing variation in CHC profiles among female, flatwing male and normal‐wing male mapping individuals. Discriminant function 1 explained 78.8% of the variance in CHC profiles between groups. Means ± 2SD are indicated by open black and white circles and lines, respectively.

We tested the consequences of the rapid invasion of *flatwing* into the *T. oceanicus* genome for other relevant phenotypes by focusing on a distinct, close‐range sexual signaling modality that operates alongside acoustic signaling in field crickets. CHCs are long‐chain, waxy molecules expressed on insect cuticles. CHCs are thought to have evolved for dessication resistance, and they tend to be expressed as a bouquet of numerous individual hydrocarbon compounds. *Teleogryllus oceanicus* CHCs are sexually dimorphic and function as sexual signals during male and female mate choice (Tregenza and Wedell 1997; Thomas and Simmons 2009, 2010), and they have been found to vary between flatwing and normal‐wing male crickets (Simmons et al. 2014). We characterized the CHC profiles of F_3_ mapping individuals, all of which were raised in a common garden environment, by extracting their CHCs and using gas chromatography–mass spectrometry (GC–MS) to measure the abundance of 26 individual compounds (Figure 3A and Table S2). By performing dimension reduction using PCA of the CHC profiles, we first established that, in our mapping population, males carrying *flatwing* showed noticeably different CHC profiles from *normal‐wing* males (Fig. 3B) (multivariate analysis of variance on six PC with eigenvalues >1 describing male CHC blends: *F*_6,191_ = 29.769, *P* < 0.001) (Table S15).

QTL analysis was performed on the first six CHC PCs using the same set of male mapping individuals, to determine whether *flatwing*‐associated variation in male CHC profiles mapped to identifiable genomic regions. The putative X chromosome, LG1, was of particular interest, because we hypothesized that the striking variation between CHC profiles of flatwing and normal‐wing males could be due to pleiotropy or hitchhiking associated with *flatwing*. Genetic mapping of CHCs was performed blind to male morphotype. PC1, which explained over one‐third of the variance in male CHC profiles, mapped to a ca. 2.5 cM region strongly co‐localized with *flatwing* (Fig. 3C). PCs 4 and 6 also had co‐localizing peaks (Fig. S8). As dimension reduction for CHCs can obscure phenotypic patterns in the original individual chemical compounds, we mapped each of the 26 compounds separately. Of these, nine showed significant peaks co‐localizing with *flatwing* (Fig. 3D). We recovered no autosomal QTL peaks for PCs 1–6, and only one QTL peak for one compound on one autosome (compound 11, 7‐C31ene, on LG8). However, the latter peak was weakly supported, with only a single marker showing an association at FDR‐corrected *P* < 0.001.

We interrogated genes on scaffolds under the CHC QTL peaks following a similar procedure used to produce the *flatwing* candidate gene set (Table S16). Of 55 protein‐coding genes, a subset of six was implicated for every CHC trait with a significant QTL peak, and these six genes were also present in the *flatwing* candidate gene set. These are strong candidates for testing for any pleiotropic or linked effects of evolved acoustic sexual signal loss on chemical sexual signals. Our final step was to explore the nature of the phenotypic shift in flatwing male CHC profiles. It is unknown how flatwing males’ profiles compare to those of females (Simmons et al. 2014), but given the generally feminizing effect of *flatwing* on male wing morphology, we predicted that flatwing males’ CHC profiles would also be feminized. We compared them to the profiles of normal‐wing males and females using DFA on profiles from all three groups. Discriminant function 1 (eigenvalue = 2.526) explained 78.8% of the variance, and indicated that flatwing male crickets’ CHC profiles are strongly feminized (Fig. 3E). Their CHCs appear to be correspondingly less attractive to females (Gray et al. 2014).

### CONCLUSIONS

Factors constraining rapid adaptation will be increasingly important to evaluate as natural populations are placed under pressure from climate change, anthropogenic disturbances, and the application of biological control agents (Tomasetto et al. 2017). The rapid emergence and spread of flatwing crickets on Kauai is a textbook example of rapid adaptation in the wild (Dugatkin 2008). Previous work on this population of crickets has found differences in the level of phenotypic plasticity, gene expression, and other reproductive characteristics such as male testis size between male *normal‐wing* and *flatwing* genotypes (Bailey et al. 2010; Pascoal et al. 2016a; Pascoal et al. 2018), and our present findings reveal the genomic footprint of strong, associated effects on sexual signaling in an entirely different sensory channel. These consequences of rapid adaptive trait loss are early‐acting, genome‐wide, and impact a range of important fitness traits. The suite of characters affected in flatwing crickets is reminiscent of feminized alternative male morphs in ruff (*Calidris pugnax*) in which an extensive supergene in a large inversion controls size, ornament and behavioral traits simultaneously (Kupper et al. 2016), and in feminized bulb mites (Joag et al. 2016). It is surprising that an evolved loss of function variant could lead to such similarly wide‐ranging phenotypic impacts so quickly, and yet still be adaptive.

Examples of rapid adaptive evolution are well known, from industrial melanism in Kettlewell's peppered moths (*Biston betuliaria*) (van't Hof et al. 2011) to insecticide resistance in mosquitoes (Ranson et al. 2002), but in general, adaptation has been thought to be mutation‐limited with negative pleiotropic consequences ascribed a prominent impeding role (Barrett and Schluter 2008). Strikingly, at least three additional independent male song‐loss variants in the Hawaiian cricket system have been recently described: a less‐feminized version of flatwing on the island of Oahu (Pascoal et al. 2014), plus “curly‐wing” and “short‐wing” crickets on Oahu and the Big Island, respectively (Rayner et al. 2019a). All of these adaptations involve morphological disruption to forewings, and their proliferation under fly selection hints that episodes of rapid adaptive evolution might be more likely when adaptation can proceed via secondary trait loss rather than gain. Future work would benefit from investigating whether the indirect genomic consequences of adaptive trait‐loss mutations are less detrimental than those of mutations underlying trait gain. The genomic signature of recent, abrupt song loss in Hawaiian crickets uniquely illustrates how genetic variants exerting large effects and accompanying widespread, associated consequences on gene expression and other phenotypes can invade genomes in the wild. Our results raise the possibility that disruptive genomic consequences of new genetic variants might place fewer constraints on rapid adaptation than previously appreciated.

Associate Editor: J. Mank

## Supporting information


**Figure S1**. Workflow diagram of repeat annotation (top) and gene prediction (bottom) pipelines.
**Figure S2**. Cross design for linkage and QTL mapping.
**Figure S3**. Histograms illustrating the identification of a CHC sample outlier. Sample B7, a normal‐wing male, is indicated by the enlarged red dot in each plot.
**Figure S4**. Reconstructed pseudomolecule for LG1 (putative X chromosome) using LOD5‐supported markers.
**Figure S5**. Venn diagram of genes predicted for *T. oceanicus* using different methods.
**Figure S6**. Proportions of eight major categories of transposable elements detected in the *T. oceanicus* genome.
**Figure S7**. MA plot of thoracic genes DE between *T. oceanicus* embryos that were homozygous for *flatwing* versus *normal‐wing*.
**Figure S8**. Genomic regions associated with different principal components describing male CHC profiles.
**Table S1**. Allele replacement table for identifying the X chromosome in the *T. oceanicus* linkage map.
**Table S2**. Identification of *Teleogryllus oceanicus* cuticular hydrocarbon profile peaks using gas chromatography–mass spectrometry.
**Table S3**. *T. oceanicus* genome metrics.
**Table S4**. List of chimeric scaffolds identified and corrected in the *T. oceanicus* genome.
**Table S5**. Summary statistics describing scaffold anchoring on *T. oceanicus* LOD5 linkage map markers.
**Table S6**. Functional annotation of *T. oceanicus* genes.
**Table S7**. Distribution of repetitive elements for each scaffolded *T. oceanicus* linkage group.
**Table S8**. Comparison of gene features among ten insect species.
**Table S9**. Transposable element classification in *T. oceanicus* contrasted with three published genomes.
**Table S10**. Thoracic gene expression variation between embryonic crickets carrying *flatwing* versus *normal‐wing* genotypes.
**Table S11**. GO analysis of thoracic DEGs between embryos carrying *flatwing* versus *normal‐wing* genotypes.
**Table S12**. KEGG pathway enrichment of thoracic DEGs between embryos carrying *flatwing* vs. *normal‐wing* genotypes.
**Table S13**. Top candidate genes associated with flatwing.
**Table S14**. GO analysis of candidate flatwing‐associated genes (CGs).
**Table S15**. Principal components with eigenvalues >1 from PCA on male CHC profiles.
**Table S16**. Candidate gene set associated with each CHC phenotype (individual or principal component) that yielded a significant QTL on the putative X (LG1), with the flatwing QTL for comparison.
