# Supplementary material for: Field cricket genome reveals the footprint of recent, abrupt adaptation in the wild
Source: Evol Lett. 2019 Dec 19;4(1):19–33. doi: 10.1002/evl3.148 (PMC7006468; doi:10.1002/evl3.148)
Supplement: Supplementary file 1 — Figure S1. Workflow diagram of repeat annotation (top) and gene prediction (bottom) pipelines. Figure S2. Cross design for linkage and QTL mapping. Figure S3. Histograms illustrating the identification of a CHC sample outlier. Sample B7, a normal‐wing male, is indicated by the enlarged red dot in each plot. Figure S4. Reconstructed pseudomolecule for LG1 (putative X chromosome) using LOD5‐supported markers. Figure S5. Venn diagram of genes predicted for T. oceanicus using different methods. Figure S6. Proportions of eight major categories of transposable elements detected in the T. oceanicus genome. Figure S7. MA plot of thoracic genes DE between T. oceanicus embryos that were homozygous for flatwing versus normal‐wing. Figure S8. Genomic regions associated with different principal components describing male CHC profiles. Table S1. Allele replacement table for identifying the X chromosome in the T. oceanicus linkage map. Table S2. Identification of Teleogryllus oceanicus cuticular hydrocarbon profile peaks using gas chromatography–mass spectrometry. Table S3. T. oceanicus genome metrics. Table S4. List of chimeric scaffolds identified and corrected in the T. oceanicus genome. Table S5. Summary statistics describing scaffold anchoring on T. oceanicus LOD5 linkage map markers. Table S6. Functional annotation of T. oceanicus genes. Table S7. Distribution of repetitive elements for each scaffolded T. oceanicus linkage group. Table S8. Comparison of gene features among ten insect species. Table S9. Transposable element classification in T. oceanicus contrasted with three published genomes. Table S10. Thoracic gene expression variation between embryonic crickets carrying flatwing versus normal‐wing genotypes. Table S11. GO analysis of thoracic DEGs between embryos carrying flatwing versus normal‐wing genotypes. Table S12. KEGG pathway enrichment of thoracic DEGs between embryos carrying flatwing vs. normal‐wing genotypes. Table S13. Top candidate genes associated with fla [file EVL3-4-19-s001.pdf]

Supporting Information for:

Field Cricket Genome Reveals the Footprint of Recent, Abrupt Adaptation in the Wild

Sonia Pascoal[1†], Judith E. Risse[2,3†], Xiao Zhang[4†], Mark Blaxter[5,6], Timothee Cezard[5], Richard J. Challis[5], Karim Gharbi[5,7], John Hunt[8,9], Sujai Kumar[5], Emma Langan[5,10], Xuan Liu[11], Jack G. Rayner[4], Michael G. Ritchie[4], Basten L. Snoek[12,13], Urmi Trivedi[5], Nathan W. Bailey[4,14]

[1]*Department of Zoology, University of Cambridge, CB2 3EJ, United Kingdom.*
[2] *Bioinformatics, Department of Plant Sciences, Wageningen University & Research, 6708 PB Wageningen, The Netherlands.*
[3]*Animal Ecology, Netherlands Institute of Ecology, PO Box 50, 6700 AB Wageningen, The Netherlands.*
[4]*School of Biology, University of St Andrews, St Andrews, Fife KY16 9TH, United Kingdom.*
[5]*Edinburgh Genomics, University of Edinburgh, Edinburgh EH9 3JT, United Kingdom.*
[6]*Institute of Evolutionary Biology, University of Edinburgh, Edinburgh EH9 3JT, United Kingdom.*
[7]*Earlham Institute, Norwich Research Park, Norwich NR4 7UZ, United Kingdom.*
[8]*School of Science and Health and the Hawkesbury Institute for the Environment, Western Sydney University, Penrith, NSW 2751, Australia.*
[9]*Centre for Ecology and Conservation, University of Exeter, Cornwall Campus, Penryn TR10 9FE, United Kingdom.*
[10]*School of Environmental Sciences, University of East Anglia, Norwich Research Park, Norwich NR4 7UZ, United Kingdom.*
[11]*Centre for Genomic Research, University of Liverpool, Liverpool L69 7ZB, United Kingdom.*
[12]*Theoretical Biology and Bioinformatics, Utrecht University, Padualaan 8, 3584 CH Utrecht, The Netherlands.*
[13]*Terrestrial Ecology, Netherlands Institute of Ecology, PO Box 50, 6700 AB Wageningen, The Netherlands.*
[14]*E-mail: nwb3@st-andrews.ac.uk*

† Contributed equally

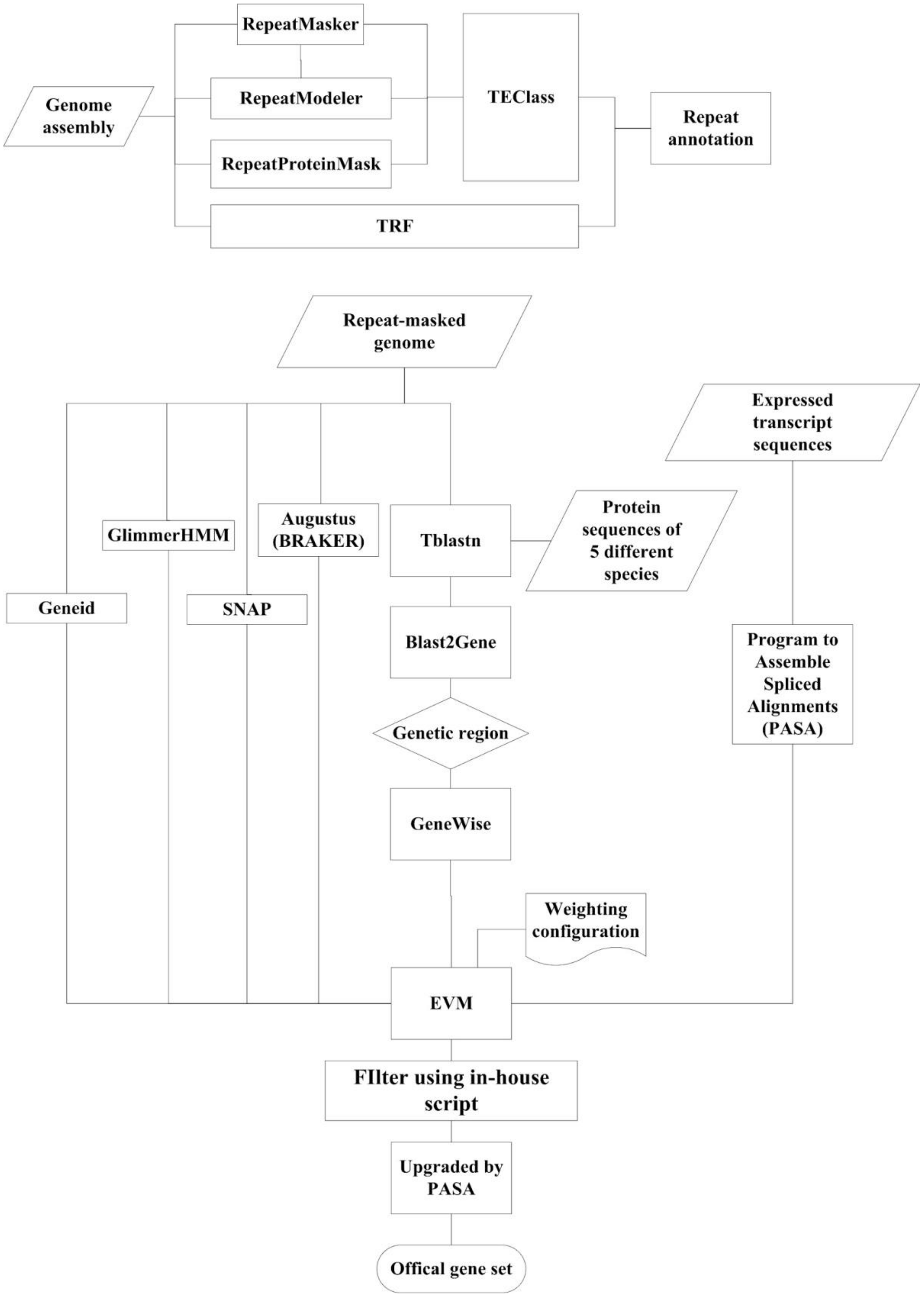

Figure S1. Workflow diagram of repeat annotation (top) and gene prediction (bottom) pipelines. Description of packages and parameters plus references are provided in the Methods section.

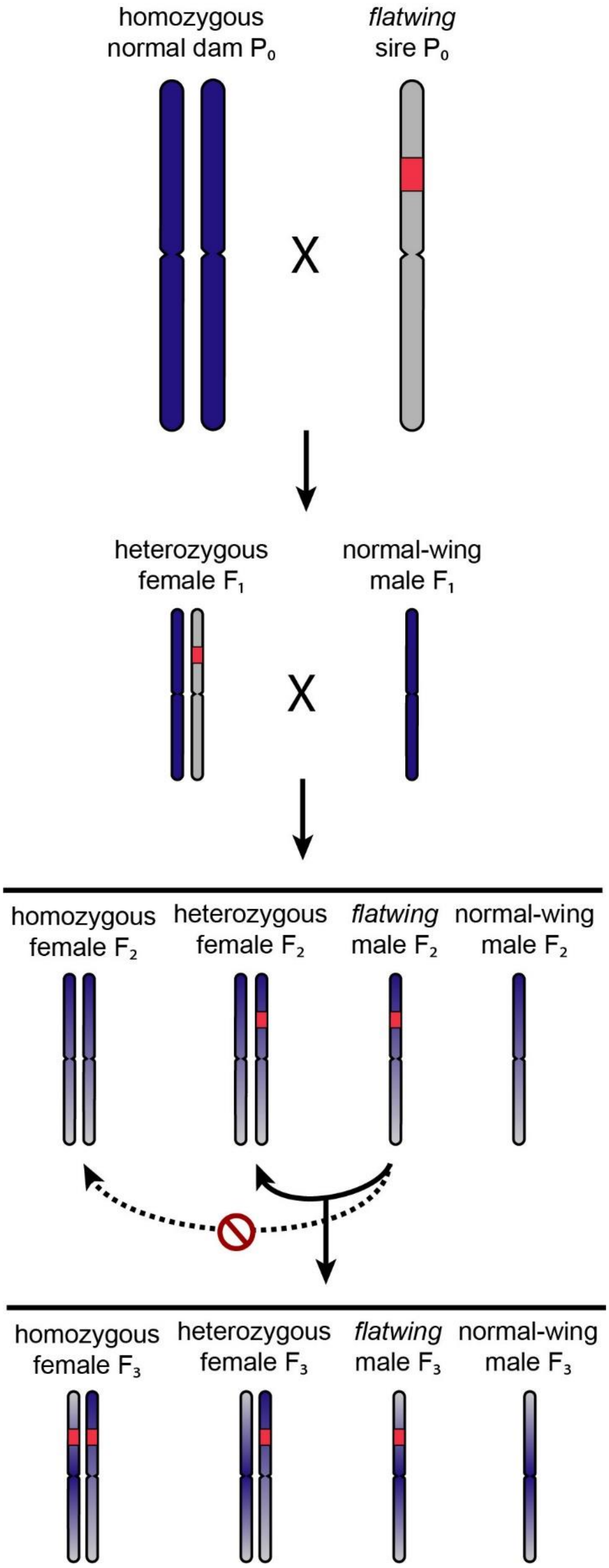

Figure S2. Cross design for linkage and QTL mapping. *Flatwing* segregates as a single-locus X-linked trait, so only X chromosomes are illustrated. A hypothetical *flatwing* locus is shaded in red. Females and males are XX/XO in *T. oceanicus*, so we performed three generations of crossing to enhance our ability to map *flatwing*. Homozygous *normal-wing* dams were obtained from a laboratory population of the same species originally derived from a population that has never contained *flatwing* (dark blue chromosomes). In the parental generation, these *normal-wing* dams were crossed to *flatwing* sires from Kauai (light grey chromosome, with hypothetical *flatwing* locus shaded in red). Dam genotypes were undetectable at generation F_2 due to *flatwing's* sex-limited expression, so only full-sib crosses for which the flatwing male phenotype segregated in the subsequent F_3 generation were retained for phenotyping and QTL mapping (solid arrows).

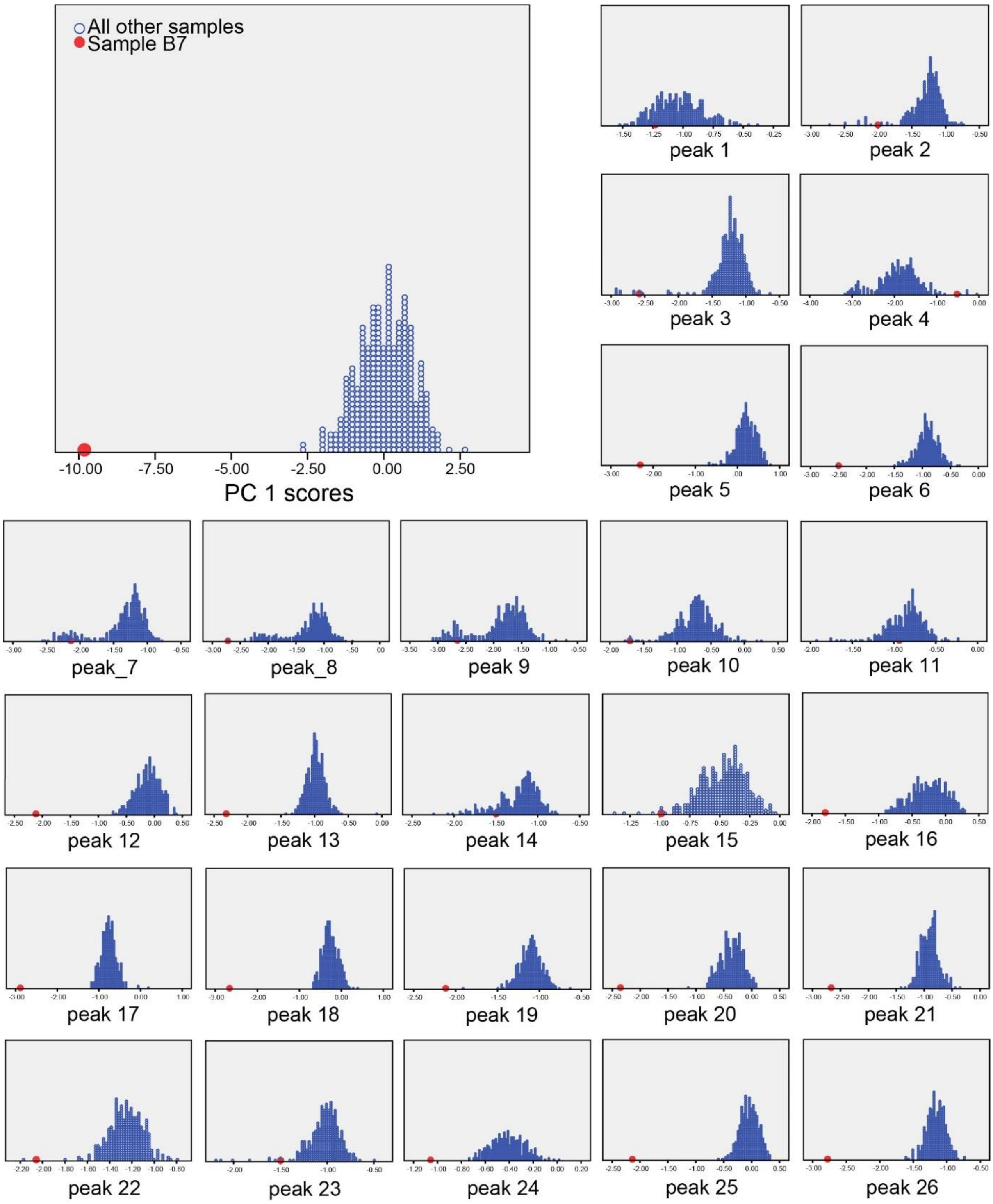

Figure S3. Histograms illustrating the identification of a CHC sample outlier. Sample B7, a normal-wing male, is indicated by the enlarged red dot in each plot. The sample was observed on visual inspection to deviate substantially from the distribution of principal component 1 scores for all other mapping individuals. Further inspection revealed this also to be the case in the majority of cases when the sample was assessed for each CHC peak individually. It was thus excluded from further analysis.

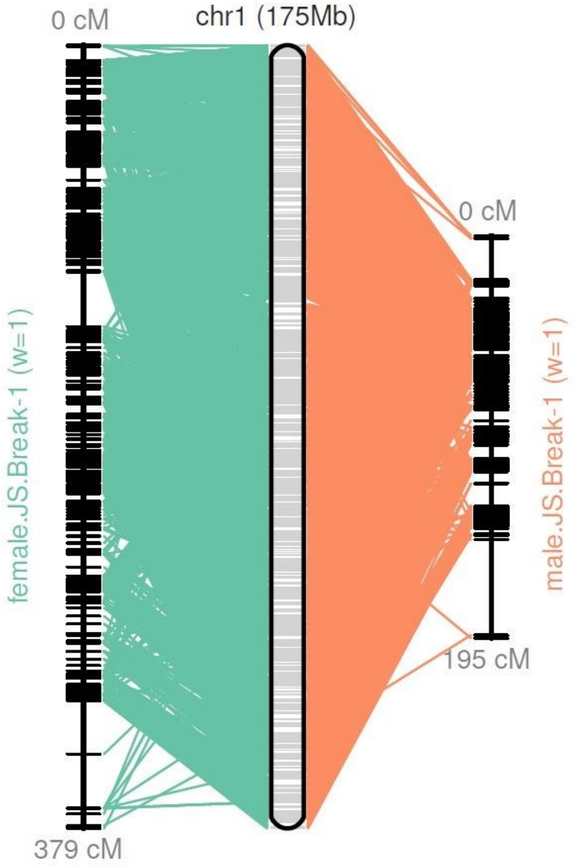

Figure S4. Reconstructed pseudomolecule for LG1 (putative X chromosome) using LOD5-supported markers. Lines connect the physical positions of markers on the pseudomolecule to female and male map positions (left and right, respectively).

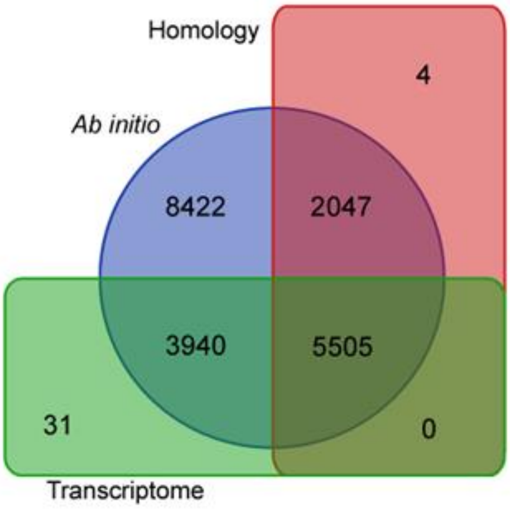

Figure S5. Venn diagram of genes predicted for *T. oceanicus* using different methods. Counts refer to the gene set that was obtained prior to final upgrade and filtering using PASA[73], so the total gene number above is slightly higher than the final gene set. A detailed description of each pipeline is presented in the Methods.

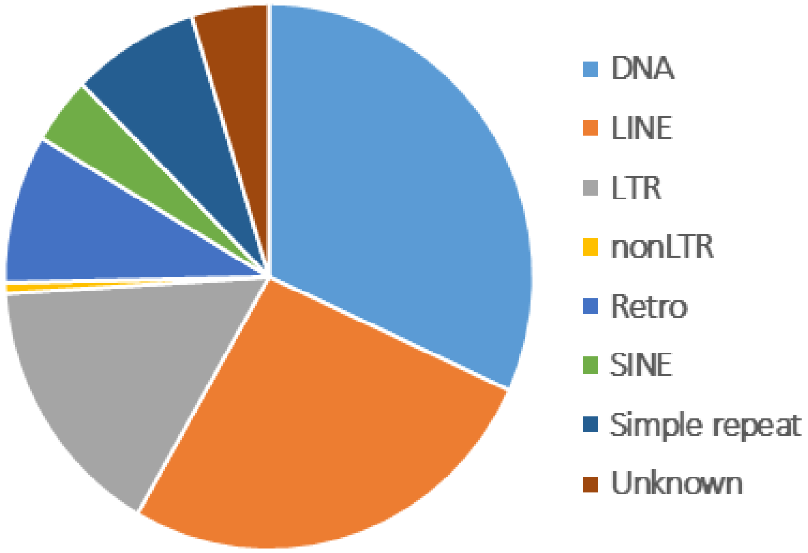

Figure S6. Proportions of eight major categories of transposable elements detected in the *T. oceanicus* genome.

DNA = DNA transposons
LTR = long terminal repeats
LINE = long interspersed nuclear elements
SINE = short interspersed nuclear elements
Retro = retrotransposon

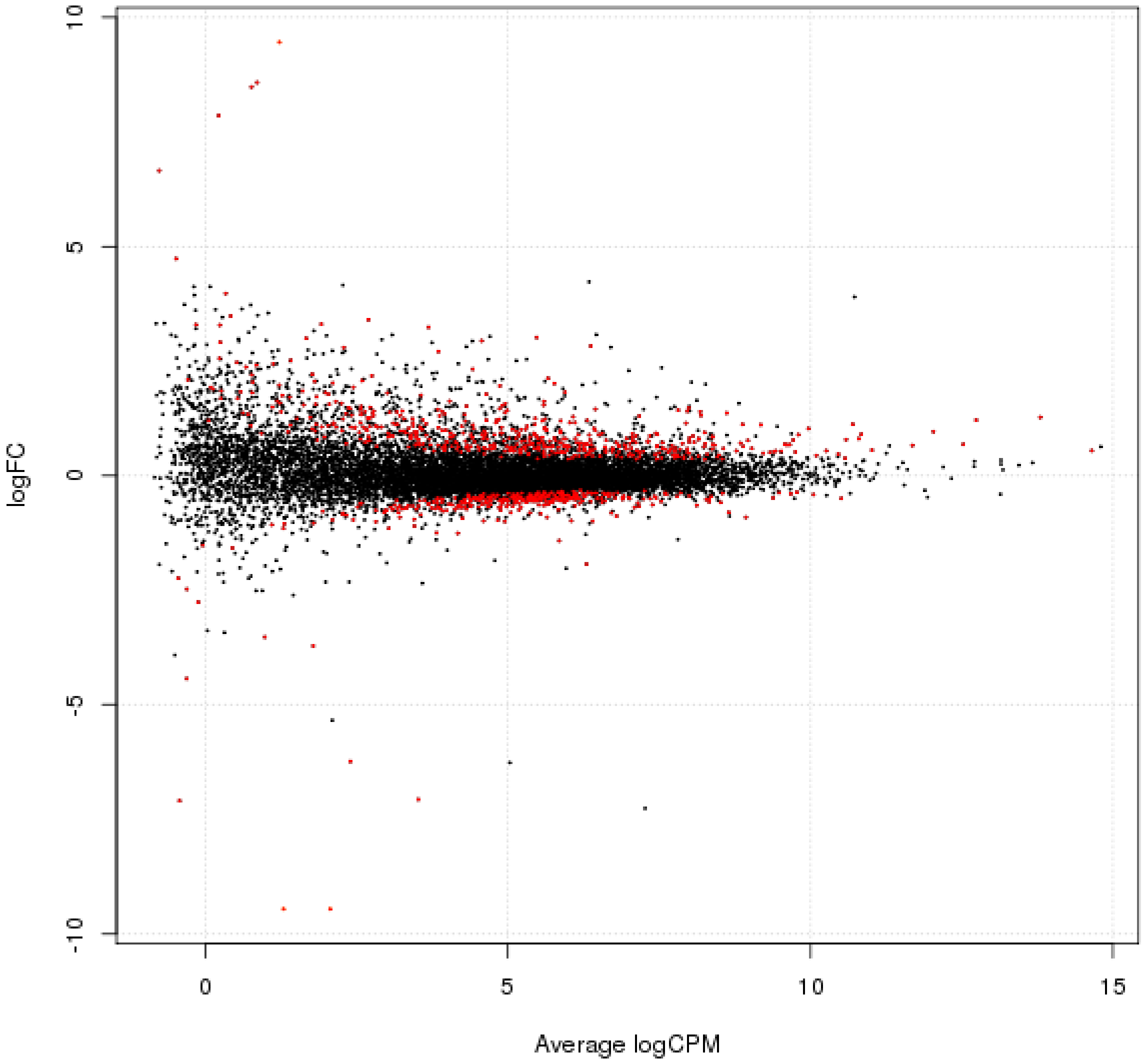

Figure S7. MA plot of thoracic genes DE between *T. oceanicus* embryos that were homozygous for *flatwing* vs. *normal-wing*. Red points indicate significantly differentially-expressed genes after filtering (see Methods), with positive values on the y-axis indicating genes downregulated in *flatwing* samples compared to *normal-wing* samples, and negative values indicating genes that are upregulated in *flatwing* samples. Log scales are base 2.

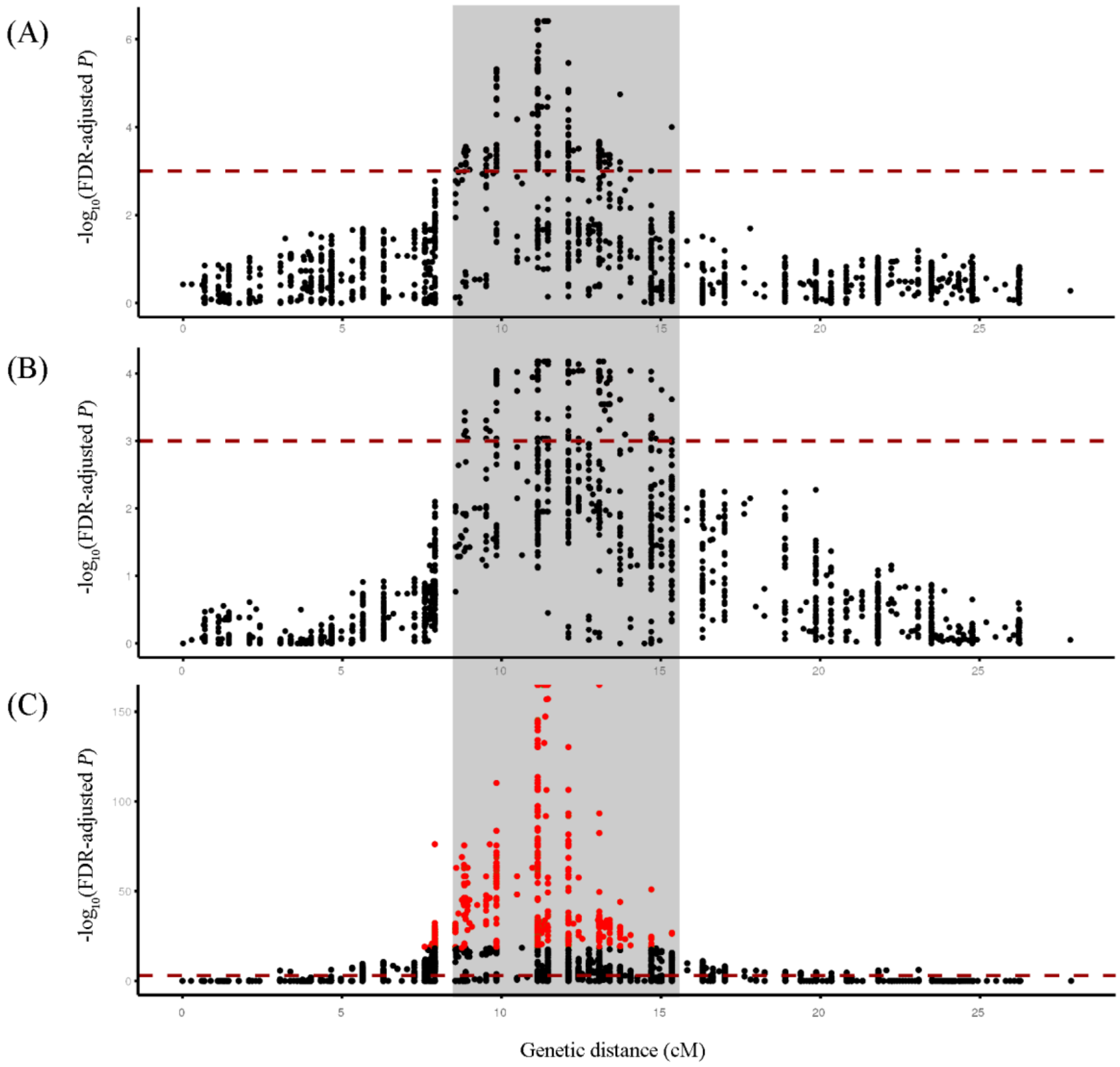

Figure S8. Genomic regions associated with different principal components describing male CHC profiles. Manhattan plot for LG1 (putative X chromosome) showing (*A*) the PC4-associated QTL, (*B*) PC6-associated QTL and (*C*) the *flatwing* QTL for comparison. The horizontal dashed lines indicate FDR-corrected significance threshold of P < 0.001, and the top 1% most significant flatwing-associated QTL markers are plotted in red in **C**. The light grey rectangle spans the genetic region in which flatwing-associated markers and CHC principal component-associated markers co-localize.

Table S1. Allele replacement table for identifying the X chromosome in the *T. oceanicus* linkage map

Dam	Sire	Sire assumed	Sire rewrite	male current	male rewrite	male current	male rewrite
**0/1**	0/0	0/.	0/2	0/0	0/2	1/1	1/2
**0/1**	1/1	1/.	1/2	0/0	0/2	1/1	1/2
**0/1**	2/2	2/.	NA	-	-	-	-
**0/2**	0/0	0/.	0/1	0/0	0/1	2/2	1/2
**0/2**	1/1	1/.	NA	-	-	-	-
**0/2**	2/2	2/.	2/1	0/0	0/1	2/2	2/1
**1/2**	0/0	0/.	NA	-	-	-	-
**1/2**	1/1	1/.	1/3	1/1	1/3	2/2	2/3
**1/2**	2/2	2/.	2/3	1/1	1/3	2/2	2/3

Table S2. Identification of *Teleogryllus oceanicus* cuticular hydrocarbon profile peaks using gas chromatography - mass spectrometry

**Peak**[a]	**Kováts retention index**[b]	**Identification**	**Diagnostic ions**
standard		pentadecane	
1	2840	$4MeC_{28}$	365, 71
2	2893	$10MeC_{28}$	281, 155
3	2910	$13MeC_{29}$	252, 196
4	3028	$C_{30:1}$	434
5	3043	$4MeC_{30}$	436, 393, 71
6	3064	7,x-diMeC_{30}	365, 112
7	3075	unidentified	
8	3086	$C_{31:1}$	434
9	3110	$11MeC_{31}$	308, 168
10	3119	7,8MeC_{31}	364, 112
11	3130	7-C_{31}ene	434, 528[c], 145[c], 383[c]
12	3142	$C_{31:1}$	434
13	3152	$C_{31:2}$	432
14	3161	$C_{31:2}$	432
15	3174	$C_{31:2}$	432
16	3188	$C_{31:2}$	432
17	3242	$4MeC_{32}$	421, 71
18	3252	unidentified	
19	3268	$C_{32:2}$	446
20	3288	$C_{33:1}$	462
21	3331	$C_{33:1}$	462
22	3347	$C_{33:2}$	460
23	3355	$C_{33:2}$	460
24	3365	$C_{33:2}$	460
25	3379	3,x-$C_{33:2}$	460, 647[c], 89[c]
26	3391	$C_{33:2}$	460

[a] Peak identification is based on Table S4 of Pascoal et al. (2016) (Pascoal et al. 2016b), reproduced here.
The 26 quantified peaks are presented in sequential order in the table and in Fig. 3A of the main text.
[b] Calculation of the Kováts retention index using *n*-alkane standards (C_7 - C_{40}) is described in Majlát et al. (1974) (Majlat et al. 1974)
[c] Ions used when identifying with dimethyl-disulphide derivation

Table S3. *T. oceanicus* genome metrics

Maximum scaffold length (bp)	2,637,780	
Complete BUSCO (Ref total = 1,066)	1,001	
% complete BUSCO (genome)	93.9%	
% complete BUSCO (gene set)[a]	83.2%	
**Scaffold metrics**	**All contigs**	**Contigs > 1,000 bp**
Total bases (gb)	2,045,067,382	2,044,651,628
N50 (bp)	62,615	62,685
Sequences in N50	6,139	6,136
GC content (%)	40	40
Mean scaffold length (bp)	10,335	10,355
**Sequencing library yields**	**Read pairs**	**Yield (GB)**
Illumina TruSeq 180	277,076,641	55.42
Illumina TruSeq 300	243,927,180	48.79
Illumina TruSeq 600	238,275,727	47.66
Illumina TruSeq Nano 350	70,959,741	14.19
Illumina TruSeq Nano 550	63,415,263	12.68
Illumina Nextera mate-pair	229,431,023	45.89
PacBio RSII	5,771,779	21.74

[a] Final gene number identified in Table S8

Table S4. List of chimeric scaffolds identified and corrected in the *T. oceanicus* genome

Chimeric scaffold	Coordinates		Corrected scaffold	Linkage group
	Start	Stop		
Contig112_pilon	1	280481	Contig112_pilon.1	LG13
Contig112_pilon	285560	415702	Contig112_pilon.2	LG4
Contig115174_pilon	1	4504	Contig115174_pilon.1	LG3
Contig115174_pilon	4505	9682	Contig115174_pilon.2	LG5
Contig11656_pilon	1	69510	Contig11656_pilon.1	LG3
Contig11656_pilon	71880	189927	Contig11656_pilon.2	LG2
Contig122717_pilon	1	791	Contig122717_pilon.1	LG12
Contig122717_pilon	792	1738	Contig122717_pilon.2	LG13
Contig12684_pilon	1	94718	Contig12684_pilon.1	LG14
Contig12684_pilon	94719	233653	Contig12684_pilon.2	LG16
Contig157093_pilon	1	21374	Contig157093_pilon.1	LG1
Contig157093_pilon	21375	29205	Contig157093_pilon.2	LG2
Contig16901_pilon	1	14926	Contig16901_pilon.1	LG16
Contig16901_pilon	18394	186701	Contig16901_pilon.2	LG11
Contig17374_pilon	1	391141	Contig17374_pilon.1	LG11
Contig17374_pilon	392712	614243	Contig17374_pilon.2	LG3
Contig19418_pilon	1	216097	Contig19418_pilon.1	LG10
Contig19418_pilon	220070	446236	Contig19418_pilon.2	LG12
Contig24478_pilon	1	10308	Contig24478_pilon.1	LG19
Contig24478_pilon	13057	232760	Contig24478_pilon.2	LG13
Contig25912_pilon	1	178241	Contig25912_pilon.1	LG12
Contig25912_pilon	180760	432977	Contig25912_pilon.2	LG11
Contig3004_pilon	1	113166	Contig3004_pilon.1	LG10
Contig3004_pilon	113846	201707	Contig3004_pilon.2	LG1
Contig30253_pilon	1	75616	Contig30253_pilon.1	LG6
Contig30253_pilon	75924	107012	Contig30253_pilon.2	LG10
Contig30890_pilon	1	42473	Contig30890_pilon.1	LG7

Contig30890_pilon	42474	357127	Contig30890_pilon.2	LG4
Contig32501_pilon	1	79400	Contig32501_pilon.1	LG8
Contig32501_pilon	81158	104315	Contig32501_pilon.2	LG5
Contig34163_pilon	1	276874	Contig34163_pilon.1	LG14
Contig34163_pilon	278116	477845	Contig34163_pilon.2	LG8
Contig34793_pilon	1	35174	Contig34793_pilon.1	LG13
Contig34793_pilon	35175	226445	Contig34793_pilon.2	LG4
Contig37346_pilon	1	181531	Contig37346_pilon.1	LG1
Contig37346_pilon	185444	510953	Contig37346_pilon.2	LG5
Contig44873_pilon	1	96939	Contig44873_pilon.1	LG3
Contig44873_pilon	100500	540225	Contig44873_pilon.2	LG2
Contig53403_pilon	1	162159	Contig53403_pilon.1	LG1
Contig53403_pilon	163594	231179	Contig53403_pilon.2	LG12
Contig6264_pilon	1	582129	Contig6264_pilon.1	LG1
Contig6264_pilon	582130	671930	Contig6264_pilon.2	LG16
Contig6264_pilon	675095	875693	Contig6264_pilon.3	LG1
Contig67999_pilon	1	75111	Contig67999_pilon.1	LG8
Contig67999_pilon	80918	230728	Contig67999_pilon.2	LG16
Contig7355_pilon	1	31398	Contig7355_pilon.1	LG4
Contig7355_pilon	31626	89218	Contig7355_pilon.2	LG7

Table S5. Summary statistics describing scaffold anchoring on *T. oceanicus* LOD5 linkage map markers

	Anchored	Oriented	Unplaced
Markers (unique)	104,713	88,665	741
Markers per Mb	143.9	154.5	0.6
Scaffolds	8,169	5,997	189,726
Scaffolds with 1 marker	686	0	187
Scaffolds with 2 markers	587	471	63
Scaffolds with 3 markers	578	368	37
Scaffolds with >=4 markers	6,318	5,158	50
Total bases	727,468,034 (35.6%)	573,790,325 (28.1%)	1,317,555,539 (64.4%)

Table S6. Functional annotation of *T. oceanicus* genes

		Number	Proportion of all genes (%)
**Total**		19,157	-
**Annotated**	InterPro	12,318	64.3
	Swissprot	11,754	61.4
	TrEMBL	13,999	73.1
	NR	13,989	73.0
	Gene Ontology	13,177	68.7
	KEGG	9,579	50.0
	All annotated	14,367	75.0
**Unknown**		4,790	25.0

Table S7. Distribution of repetitive elements for each scaffolded *T. oceanicus* linkage group

Linkage group	Transposable elements (kb)	%	Rank[a]	Tandem repeats (kb)	%	Rank[a]	Combined (kb)	%	Rank[a]
LG1	65992	38.6	8	1832	1.1	20	67823	39.7	11
LG2	26518	35.7	17	1123	1.5	14	27641	37.2	17
LG3	12908	36.3	14	572	1.6	13	13480	37.9	14
LG4	16514	42.0	2	848	2.2	4	17362	44.2	2
LG5	10253	36.2	15	500	1.8	11	10753	37.9	15
LG6	23187	35.0	18	925	1.4	17	24113	36.4	18
LG7	17533	36.3	13	936	1.9	8	18469	38.2	13
LG8	12770	38.8	6	617	1.9	7	13387	40.7	6
LG9	9952	38.2	9	659	2.5	2	10611	40.7	7
LG10	9359	38.2	10	545	2.2	5	9904	40.5	8
LG11	18920	34.1	19	804	1.5	15	19724	35.6	19
LG12	4850	42.7	1	297	2.6	1	5148	45.4	1
LG13	12684	37.4	12	624	1.8	10	13308	39.2	12
LG14	12629	36.2	16	483	1.4	16	13112	37.6	16
LG15	1298	41.7	3	53	1.7	12	1351	43.5	3
LG16	6292	31.5	20	243	1.2	19	6535	32.7	20
LG17	337	39.5	5	10	1.2	18	347	40.7	5
LG18	699	38.2	11	42	2.3	3	741	40.5	9
LG19	3	27.4	21	0	0.4	21	3	27.8	21
Unplaced	526597	40.0	4	25416	1.9	6	552013	41.9	4
Total	789295	38.6	7	36531	1.8	9	825826	40.4	10

[a] Sorted by the proportion of repetitive elements in linkage groups, from most to fewest

Table S8. Comparison of gene features among ten insect species

Species	Genome assembly size (Gb)	Gene number	Single exon gene number	%	Average gene length	Average CDS length (bp)	Average exon number per transcript	Average exon length (bp)	Ref.
**Field cricket**									
Teleogryllus oceanicus	2.0	19,157	1,266	6.6	12,232	1,184	6.2	395	*this study*
**Migratory locust**									
Locusta migratoria	6.5	17,307	3,079	17.8	54,341[a]	1,160	5.8	201	(Wang et al. 2014)
**American Cockroach**									
Periplaneta americana	3.4	21,336	2,704	12.7	12,963[b]	-	4[b]	247[b]	(Li et al. 2018)
**Fruit fly**									
Drosophila melanogaster	0.1	13,689	2,761	20.2	4,261	1,621	4.0	408	(Gramates et al. 2017)[c]
**Asian long-horned beetle**									
Anoplophora glabripennis	0.7	16,200	1,194	7.4	18,596	1,744	6.6	265	(McKenna et al. 2016)
**Bed bug**									
Cimex lectularius	0.7	13,751	1,410	10.3	29,076	1,846	10.2	265	(Rosenfeld et al. 2016)
**Brown planthopper**									
Nilaparvata lugens	1.1	21,442	2,179	10.2	22,015	1,440	6.9	289	(Xue et al. 2014)
**Dampwood termite**									
Zootermopsis nevadensis	0.5	15,129	370	2.4	24,927	1,890	9.4	365	(Terrapon et al. 2014)
**Yellow fever mosquito**									
Aedes aegypti	1.3	19,585	1,158	5.9	36,583	2,144	6.4	481	(Dudchenko et al. 2017)
**Asian Tiger mosquito**									
Aedes albopictus	2.2	38,706	2,305	6.0	25,506	1,950	5.3	440	(Miller et al. 2018)

[a] Originally reported average transcript length
[b] Originally reported medians
[c] Adapted from (Wang et al. 2014)

Table S9. Transposable element classification in *T. oceanicus* contrasted with three published genomes

	T. oceanicus		***L. migratoria***[a]		***D. melanogaster***[a]		***H. sapiens***[a]	
Types	Repeat size (bp)	% of genome	Repeat size (bp)	% of genome	Repeat size (bp)	% of genome	Repeat size (bp)	% of genome
DNA	259181458	12.7	1,480,538,225	22.7	4,849,763	2.9	99,797,428	3.2
LINE	215705991	10.5	1,332,720,207	20.4	12,119,904	7.2	637,919,432	20.3
LTR	127951980	6.3	508,675,263	7.8	21,849,378	13.0	267,738,295	8.5
nonLTR	5233875	0.3	63,892,419	1.0	-	-	-	-
Retro	71828043	3.5	153,548,453	2.4	-	-	-	-
SINE	32344731	1.6	141,176,698	2.2	52,841	0.0	397,225,496	12.7
Simple repeat	63555524	3.1	13,026,240	0.2	2,733	0.0	26,240,511	0.8
Unknown	38615245	1.9	406,097,360	6.2	11,211,970	6.6	1,298,163	0.0
Total	789295269	38.6	3,840,808,141	58.9	50,785,143	30.0	1,434,373,137	46.0

DNA = DNA transposons
LTR = long terminal repeats
LINE = long interspersed nuclear elements
SINE = short interspersed nuclear elements
Retro = retrotransposon
[a] Data from (Wang et al. 2014)

Table S10. Thoracic gene expression variation between embryonic crickets carrying *flatwing* vs. *normal-wing* genotypes

**Total number of genes passing expression filtering**	10,961
**Total DE genes**	830
**Up-regulated in *flatwing***	328
**Down-regulated in *flatwing***	502
**DE genes with $log_2FC > 1$**	204
**Up-regulated in *flatwing***	25
**Down-regulated in *flatwing***	179

Table S11. GO analysis of thoracic DEGs between embryos carrying *flatwing* vs. *normal-wing* genotypes

GO	Type	Function	No. of DEGs	*P*-adj.
GO:0003824	molecular_function	catalytic activity	216	0.006
GO:0016787	molecular_function	hydrolase activity	80	0.027
GO:0044087	biological_process	regulation of cellular component biogenesis	20	0.004
GO:0051493	biological_process	regulation of cytoskeleton organization	12	0.023
GO:0090066	biological_process	regulation of anatomical structure size	12	0.009
GO:0097435	biological_process	supramolecular fiber organization	12	0.023
GO:0032535	biological_process	regulation of cellular component size	11	0.004
GO:0032956	biological_process	regulation of actin cytoskeleton organization	11	0.001
GO:0032970	biological_process	regulation of actin filament-based process	11	0.001
GO:1902903	biological_process	regulation of supramolecular fiber organization	11	0.002
GO:0043254	biological_process	regulation of protein complex assembly	10	0.035
GO:0110053	biological_process	regulation of actin filament organization	10	0.001
GO:0008064	biological_process	regulation of actin polymerization or depolymerization	8	0.006
GO:0030832	biological_process	regulation of actin filament length	8	0.006
GO:0030833	biological_process	regulation of actin filament polymerization	8	0.006
GO:0032271	biological_process	regulation of protein polymerization	8	0.015
GO:0015630	cellular_component	microtubule cytoskeleton	6	0.001
GO:0005248	molecular_function	voltage-gated sodium channel activity	5	0.004
GO:0010927	biological_process	cellular component assembly involved in morphogenesis	5	0.010
GO:0034706	cellular_component	sodium channel complex	4	0.007

Table S12. KEGG pathway enrichment of thoracic DEGs between embryos carrying *flatwing* vs. *normal-wing* genotypes

ID[a]	Pathway	No. of genes	*P*-value[b]	*P*-adj.[c]
map04520	Adherens junction	13	<0.001	0.025
map03030	DNA replication	9	0.001	0.264
map05100	Bacterial invasion of epithelial cells	9	0.007	0.408
map05130	Pathogenic Escherichia coli infection	9	0.005	0.408
map01100	Metabolic pathways	59	0.013	0.552
map04960	Aldosterone-regulated sodium reabsorption	5	0.012	0.552
map04064	NF-kappa B signaling pathway	5	0.021	0.687
map04711	Circadian rhythm - fly	3	0.024	0.715
map00230	Purine metabolism	18	0.038	0.744
map03430	Mismatch repair	5	0.035	0.744
map04111	Cell cycle - yeast	10	0.033	0.744
map04115	p53 signaling pathway	6	0.037	0.744
map04670	Leukocyte transendothelial migration	7	0.037	0.744
map04927	Cortisol synthesis and secretion	5	0.031	0.744
map03410	Base excision repair	5	0.044	0.765
map04022	cGMP-PKG signaling pathway	11	0.048	0.765
map04530	Tight junction	12	0.043	0.765

[a] Pathways describing human disease not shown

[b] Fisher's exact test

[c] FDR-corrected

Table S13. Top candidate genes associated with flatwing

Coordinates						
**Scaffold**	**Start**	**Stop**	**Gene ID**		**Description[a]**	**Source[b]**
Contig10220_pilon	78887	163134	TOT000182.1	YGD6	Zinc-type alcohol dehydrogenase-like protein C1773.06c	Top 1%
Contig11287_pilon	199864	215783	TOT001075.1	HNF4	Transcription factor HNF-4 homolog	Top 1%
Contig12423_pilon	12805	162659	TOT001854.1	OSM3	Osmotic avoidance abnormal protein 3	Top 1%
Contig12752_pilon	14326	68612	TOT002129.1	ROR1	Inactive tyrosine-protein kinase transmembrane receptor ROR1	Top 1%
Contig12919_pilon	275064	329308	TOT002239.1	CLOCK	Circadian locomotor output cycles protein kaput	Top 1% DEG
Contig13810_pilon	43734	77412	TOT002877.1	CRTAP	Cartilage-associated protein	Top 1%
Contig140_pilon	14528	100227	TOT003072.1	PP4R1	Serine/threonine-protein phosphatase 4 regulatory subunit 1	Top 1%
Contig17198_pilon	217974	328434	TOT004721.1	SCN60	Sodium channel protein 60E	Top 1%
Contig17198_pilon	398116	526355	TOT004722.1	SCN60	Sodium channel protein 60E	Top 1%
Contig17528_pilon	151965	161397	TOT004867.1	OBSTE	Protein obstructor-E	Kauai DEG
Contig17791_pilon	294998	303418	TOT005017.1	PKRA	Protein krasavietz	DEG QTL
Contig18506_pilon	5030	106086	TOT005335.1	STRN3	Striatin-3	Top 1%
Contig20777_pilon	197732	433721	TOT006213.1	COLL	Transcription factor collier	Top 1% BSA
Contig23358_pilon	266813	357642	TOT006927.1	E78C	Ecdysone-induced protein 78C (Eip78C)	Top 1%
Contig23647_pilon	61437	289082	TOT006991.1	RAVR1	Ribonucleoprotein PTB-binding 1	Top 1%
Contig24519_pilon	221508	332371	TOT007217.1	A0A167 WTZ1	Endo-1,3(4)-beta-glucanase	Top 1%
Contig24519_pilon	569981	635619	TOT007221.1	SEPIA	Pyrimidodiazepine synthase	Top 1%
Contig30320_pilon	33122	79411	TOT008755.1	PTPC1	Protein tyrosine phosphatase domain-containing protein 1	Top 1%
Contig3077_pilon	487713	492969	TOT008894.1	REXO4	RNA exonuclease 4	Top 1%
Contig31374_pilon	378769	413960	TOT009065.1	CPT2	Carnitine O-palmitoyltransferase 2, mitochondrial	Top 1%
Contig31374_pilon	461061	489320	TOT009067.1	FRM4B	FERM domain-containing protein 4B	Top 1%
Contig32190_pilon	94344	248306	TOT009274.1	RN207	RING finger protein 207	Top 1%
Contig35402_pilon	14084	125884	TOT010060.1	ABCG1	ATP-binding cassette sub-family G member 1	Top 1%
Contig37346_pilon.1	133394	180067	TOT010542.1	SCYL1	N-terminal kinase-like protein	Top 1%
Contig40107_pilon	150347	172207	TOT011176.1	THUM3	THUMP domain-containing protein 3	DEG, QTL
Contig4430_pilon	60074	108676	TOT012009.1	LAR	Tyrosine-protein phosphatase Lar	Top 1%
Contig4497_pilon	323	114981	TOT012126.1	MYO5A	Unconventional myosin-Va	Top 1%

Contig48084_pilon	4534	15580	TOT012711.1	KPEL	Serine/threonine-protein kinase pelle	Top 1%
Contig48322_pilon	73569	78934	TOT012764.1	CAH10	Carbonic anhydrase-related protein 10	Top 1%
Contig52923_pilon	4299	134158	TOT013504.1	RENT2	Regulator of nonsense transcripts 2	Top 1% DEG
Contig52923_pilon	172817	234071	TOT013505.1	NBL1	Neuroblastoma suppressor of tumorigenicity 1	Top 1%
Contig53931_pilon	135337	200203	TOT013689.1	TADBP	TAR DNA-binding protein 43	Top 1%
Contig55532_pilon	2641	6823	TOT013967.1	SOSSC	SOSSC_BOVIN	Top 1%
Contig5817_pilon	13001	94458	TOTO14395.1	A0A1B6 LWD6	Uncharacterized protein	Top 1%
Contig6025_pilon	181847	338853	TOT014693.1	PAX6	Paired box protein Pax-6	Top 1%
Contig6181_pilon	7490	15461	TOT014894.1	MYCT	Proton myo-inositol cotransporter	Top 1%
Contig6371_pilon	72321	126646	TOT015146.5	GOGA4	Golgin subfamily A member 4	Top 1%
Contig6636_pilon	248427	279756	TOT015511.1	A0A067 RPQ2	LRR domain-containing protein	Top 1%
Contig6636_pilon	332473	344815	TOT015512.1	IPR0110 11	Uncharacterized protein	Top 1%
Contig66512_pilon	19778	188429	TOT015537.1	LASP1	LIM and SH3 domain protein F42H10.3	Top 1% DEG
Contig6932_pilon	94582	114944	TOT015868.1	ABCB8	ATP-binding cassette sub-family B member 8, mitochondrial	Top 1%
Contig6932_pilon	132941	150338	TOT015869.1	APMAP	Adipocyte plasma membrane-associated protein	Top 1%
Contig7020_pilon	57223	96643	TOT015999.1	SPS1	Selenide, water dikinase	Top 1%
Contig7210_pilon	172512	326460	TOT016305.1	MYO	myoglianin	Top 1%
Contig7490_pilon	12720	16839	TOT016652.1	GCN5	Histone acetyltransferase GCN5	Top 1%
Contig8190_pilon	192630	256540	TOT017431.1	AT133	Probable cation-transporting ATPase 13A3	Top 1%
Contig82459_pilon	106133	176987	TOT017512.1	UNC89	Muscle M-line assembly protein unc-89	DEG QTL
Contig83863_pilon	1777	51856	TOT017662.1	A0A017 RSC4	Uncharacterized protein	Top 1%
Contig92683_pilon	43257	76189	TOT018508.1	A0A0T6 B8G7	Uncharacterized protein	Top 1%
Contig33215_pilon	67326	419738	TOT009518.1	PLXA4	Plexin-A4	QTL BSA
Contig43580_pilon	106377	137341	TOT011864.1	RNF41	E3 ubiquitin-protein ligase NRDP1	QTL BSA

[a] Functional descriptions and references provided in Main Text

[b] The criterion for inclusion as a top candidate was that a gene had to receive support for association with the flatwing phenotype from at least two of the following four sources:

QTL = portion of the gene was located within a 1 kb flanking region of a significantly-associated (FDR-corrected) marker in the flatwing QTL analysis

Top1% = portion of the gene was located within a 1 kb flanking region of a SNP in the top 1% of significantly-associated markers in the flatwing QTL analysis (Top1% candidates automatically received “QTL” support)

BSA = portion of the gene was located within a 1 kb flanking region of a significantly-associated marker from the previously-published bulked segregant analysis of Kauai flatwing males[7] which was also anchored to LG1

DEG = gene was significantly differentially expressed between pure-breeding *normal-wing* genotypes and *flatwing* genotypes, in embryonic thoracic tissue (track iv of Fig. 2a in Main Text)

Table S14. GO analysis of candidate flatwing-associated genes (CGs)

GO	Type	Function	No. of CGs	*P*-adj. (χ2 test)
GO:0010720	biological_process	positive regulation of cell development	3	<0.001
GO:0045597	biological_process	positive regulation of cell differentiation	3	0.002
GO:0060284	biological_process	regulation of cell development	3	0.042
GO:0003707	molecular_function	steroid hormone receptor activity	2	<0.001
GO:0009755	biological_process	hormone-mediated signaling pathway	2	0.006
GO:0030545	molecular_function	receptor regulator activity	2	0.035
GO:0043401	biological_process	steroid hormone mediated signaling pathway	2	0.002
GO:0045666	biological_process	positive regulation of neuron differentiation	2	0.040
GO:0048018	molecular_function	receptor ligand activity	2	0.022

Table S15. Principal components with eigenvalues > 1 from PCA on male CHC profiles. MANOVA[a] results examine the effect of male morph on scores for each PC (multivariate model: Wilks' λ = 0.517, $F_{6,191}$ = 29.769, p < 0.001)

Principal component	Eigenvalue	% Variance explained	$F_{1,196}$	P	R^2
1	9.408	36.18	25.885	<0.001	0.131
2	2.635	10.136	18.040	<0.001	0.092
3	2.490	9.576	21.454	<0.001	0.109
4	1.888	7.261	0.001	0.979	0.000
5	1.315	5.058	25.741	<0.001	0.131
6	1.020	3.925	4.079	0.043	0.021

[a] Two-sided tests

Table S16. Candidate gene set associated with each CHC phenotype (individual or principal component) that yielded a significant QTL on the putative X (LG1), with the flatwing QTL for comparison

Coordinates			Gene ID	Trait													Description	
				Individual Compounds									PCs			Flat-wing		
Scaffold	Start	Stop		1	5	7	8	9	12	15	18	21	1	4	6			
Contig10220_pilon	78887	163134	TOT000182.1	√	√	√	√				√	√		√	√	√	YGD6	Zinc-type alcohol dehydrogenase-like protein C1773.06c
Contig11287_pilon	199864	215783	TOT001075.1	√	√	√	√	√	√	√	√	√	√	√	√	√	HNF4	Transcription factor HNF-4 homolog
Contig12423_pilon	12805	162659	TOT001854.1	√	√	√	√	√	√	√	√	√	√	√	√	√	OSM3	Osmotic avoidance abnormal protein 3
Contig14561_pilon	436209	450946	TOT003337.1								√						CAS	Transcription factor castor
Contig16800_pilon	571706	708271	TOT004499.1								√						SMAD3	Mothers against decapentaplegic homolog 3
Contig17198_pilon	217974	328434	TOT004721.1		√										√	√	SCN60	Sodium channel protein 60E
Contig17198_pilon	398116	526355	TOT004722.1	√				√			√			√		√	SCN60	Sodium channel protein 60E
Contig17589_pilon	46372	264973	TOT004897.1							√	√						SSBP3	Single-stranded DNA-binding protein 3
Contig17589_pilon	70840	324258	TOT004898.1							√	√						ATG10	Ubiquitin-like-conjugating enzyme ATG10
Contig17791_pilon	294998	303418	TOT005017.1	√	√						√	√				√	PKRA	Protein krasavietz
Contig18309_pilon	75967	136785	TOT005266.1		√												STA5B	Signal transducer and activator of transcription 5B
Contig191692_pilon	10444	19353	TOT005602.1								√						GNAI	Guanine nucleotide-binding protein G(i) subunit alpha
Contig20777_pilon	197732	433721	TOT006213.1	√	√					√	√	√	√	√		√	COLL	Transcription factor collier
Contig23454_pilon	18213	89940	TOT006946.1		√						√						ARD17	Arrestin domain-containing protein 17
Contig23647_pilon	61437	289082	TOT006991.1	√	√	√	√				√	√		√	√	√	RAVR1	Ribonucleoprotein PTB-binding 1
Contig24519_pilon	569981	635619	TOT007221.1								√					√	SEPIA	Pyrimidodiazepine synthase
Contig27628_pilon	259968	507152	TOT008051.1								√						PROH3	Prohormone-3
Contig29117_pilon	70169	396634	TOT008443.1		√	√	√				√						CCKAR	Cholecystokinin receptor type A
Contig29877_pilon	36855	181557	TOT008655.1		√												E41LA	Band 4.1-like protein 4A
Contig3077_pilon	487713	492969	TOT008894.1	√	√	√	√	√	√	√	√	√	√	√	√	√	REXO4	RNA exonuclease 4
Contig3077_pilon	528735	564924	TOT008896.1		√												P4K2B	Phosphatidylinositol 4-kinase type 2
Contig31374_pilon	461061	489320	TOT009067.1	√	√	√	√	√	√	√	√	√	√	√	√	√	FRM4B	FERM domain-containing protein 4B
Contig32190_pilon	94344	248306	TOT009274.1	√	√	√	√				√	√		√	√	√	RN207	RING finger protein 207
Contig3429_pilon	122631	136033	TOT009790.1	√							√						A0A1B6JV12	Uncharacterized protein
Contig3536_pilon	221796	347378	TOT010046.1		√						√						GNAO	Guanine nucleotide-binding protein G(o) subunit alpha
Contig35402_pilon	14084	125884	TOT010060.1		√	√					√			√	√	√	ABCG1	ATP-binding cassette sub-family G member 1
Contig3552_pilon	175659	203623	TOT010094.1		√						√						OSBL9	Oxysterol-binding protein-related protein 9
Contig37346_pilon.1	133394	180067	TOT010542.1	√	√	√	√	√	√	√	√	√	√	√	√	√	SCYL1	N-terminal kinase-like protein
Contig40569_pilon	70324	81832	TOT011293.1	√							√						NXT1	NTF2-related export protein
Contig43774_pilon	63974	228355	TOT011905.1								√						HMCN1	Hemicentin-1
Contig4430_pilon	60074	108676	TOT012009.1	√	√	√				√	√	√	√			√	LAR	Tyrosine-protein phosphatase Lar
Contig4451_pilon	20121	45182	TOT012039.1		√												LAR4	La-related protein Larp4B
Contig48084_pilon	4534	15580	TOT012711.1		√						√					√	KPEL	Serine/threonine-protein kinase pelle
Contig48322_pilon	73569	78934	TOT012764.1								√					√	CAH10	Carbonic anhydrase-related protein 10
Contig5051_pilon	34769	44241	TOT013154.1								√						CMYA5	Cardiomyopathy-associated protein 5
Contig52923_pilon	172817	234071	TOT013505.1	√	√	√	√			√	√	√		√	√	√	NBL1	Neuroblastoma suppressor of tumorigenicity 1

Contig53931_pilon	135337	200203	TOT013689.1	√	√	√				√	√	√		√		√	TADBP	TAR DNA-binding protein 43
Contig5490_pilon	190540	238169	TOT013863.1		√						√						UNC89	Muscle M-line assembly protein unc-89
Contig5490_pilon	145320	153034	TOT013873.1								√						A0A067RCZ8	Uncharacterized protein
Contig5542_pilon	24715	684526	TOT013934.1		√						√						GALT2	Polypeptide N-acetylgalactosaminyltransferase 2
Contig55532_pilon	2641	6823	TOT013967.1		√						√					√	SOSSC	SOSS complex subunit C
Contig5817_pilon	13001	94458	TOT014395.1			√					√					√	A0A1B6LWD6	Uncharacterized protein
Contig6025_pilon	181847	338853	TOT014693.1	√	√	√	√				√	√		√	√	√	PAX6	Paired box protein Pax-6
Contig6181_pilon	7490	15461	TOT014894.1		√						√				√	√	MYCT	Proton myo-inositol cotransporter
Contig6371_pilon	72321	126646	TOT015146.5	√	√	√				√	√	√		√	√	√	GOGA4	Golgin subfamily A member 4
Contig63833_pilon	68993	231055	TOT015158.1								√						HUWE1	E3 ubiquitin-protein ligase HUWE1
Contig6636_pilon	248427	279756	TOT015511.1	√	√	√				√	√	√		√		√	A0A067RPQ2	LRR domain-containing protein
Contig66512_pilon	19778	188429	TOT015537.1	√	√					√	√	√		√		√	LASP1	LIM and SH3 domain protein F42H10.3
Contig6932_pilon	94582	114944	TOT015868.1		√						√					√	ABCB8	ATP-binding cassette sub-family B member 8, mitochondrial
Contig6932_pilon	132941	150338	TOT015869.1		√						√					√	APMAP	Adipocyte plasma membrane-associated protein
Contig7210_pilon	172512	326460	TOT016305.1	√	√	√				√	√	√	√	√		√	MYO	myoglianin
Contig745_pilon	6060	59826	TOT016600.1								√						E41L5	Band 4.1-like protein 5
Contig7490_pilon	12720	16839	TOT016652.1	√	√	√				√	√	√		√	√	√	GCN5	Histone acetyltransferase GCN5
Contig8263_pilon	12087	12734	TOT017545.1		√		√				√			√	√		TWF	Twinfilin
Contig92683_pilon	43257	76189	TOT018508.1	√	√	√	√	√	√	√	√	√	√	√	√	√	A0A0T6B8G7	Uncharacterized protein

30
